# MNetClass: a control-free microbial network clustering framework for identifying central subcommunities across ecological niches

**DOI:** 10.1128/msystems.00989-25

**Published:** 2025-11-13

**Authors:** Yihua Wang, Qingzhen Hou, Fulan Wei, Bingqiang Liu, Qiang Feng

**Affiliations:** 1 School of Mathematics, Shandong University 12589, Jinan, China; 2 Department of Biostatistics, School of Public Health, Cheeloo College of Medicine, Shandong University, Jinan, China; 3 Shandong Key Laboratory of Oral Tissue Regeneration, Shandong Engineering Laboratory for Dental Materials and Oral Tissue Regeneration, Shandong Provincial Clinical Research Center for Oral Diseases, Department of Human Microbiome, School and Hospital of Stomatology, Cheeloo College of Medicine, Shandong University, Jinan, China; 4 Shandong University-BOP Joint Oral Microbiome Laboratory, Jinan, China; Katholieke Universiteit Leuven, Leuven, Belgium

**Keywords:** microbiome, network analysis, clustering, random walk algorithm, rank-sum ratio–entropy weight evaluation model

## Abstract

Investigating microbiome subnetworks and identifying central microbes in specific ecological niches is a critical issue in human microbiome studies. Traditional methods typically require control samples, limiting the ability to study microbiomes at distinct body sites without matched controls. Moreover, some clustering methods are not well-suited for microbial data and fail to identify central subcommunities across ecological niches after clustering. In this study, we present MNetClass, a novel microbial network clustering analysis framework. It utilizes a random walk algorithm and a rank-sum ratio–entropy weight evaluation model to classify key subnetworks and identify central microbes at any body site, without the need for control samples. We demonstrate its capabilities on both simulated and real microbiome data sets. Simulation results indicate that MNetClass outperforms current unsupervised microbial clustering methods. In applied case studies, the analysis of microbiome data from five distinct oral sites revealed site-specific microbial communities. Furthermore, MNetClass demonstrated superior predictive performance on cross-cohort Autism Spectrum Disorder data and identified age-related microbial communities across different oral sites, underscoring its broad applicability in microbiome research.

**IMPORTANCE:**

MNetClass provides a valuable tool for microbiome network analysis, enabling the identification of key microbial subcommunities across diverse ecological niches. Implemented as an R package (https://github.com/YihuaWWW/MNetClass), it offers broad accessibility for researchers. Here, we systematically benchmarked MNetClass against existing microbial clustering methods on synthetic data using various performance metrics, demonstrating its superior efficacy. Notably, MNetClass operates without the need for control groups and effectively identifies central microbes, highlighting its potential as a robust framework for advancing microbiome research.

## INTRODUCTION

The human body hosts a diverse microbial community that, alongside tissues and organs, forms an essential part of the organism. These microbes are crucial for maintaining health (1, 2). Due to the diverse physiological characteristics and microenvironments, the composition of microorganisms differs in each body site (3). Additionally, certain microbes show site-specific preferences, and their presence in non-native locations is linked to an increased risk of various diseases (4). For instance, *Fusobacterium nucleatum*, commonly found in the oral cavity, has been suggested to be involved in colorectal cancer upon translocation to the intestine (5). Similarly, *Porphyromonas gingivalis*, a key pathogen in periodontitis, has been associated with esophageal cancer when detected in the esophagus (6). Microbial taxa across different body sites interact to form distinct subcommunities, often participating in shared metabolic pathways or host signaling processes, which are crucial for maintaining both microbial stability and host physiological functions (7). For example, the phyla *Firmicutes* and *Bacteroidetes* dominate the gut microbiota, where their ratio remains relatively stable in healthy individuals, though an increased *Firmicutes*-to-*Bacteroidetes* ratio has been consistently linked to obesity (8). In the oral cavity, *Streptococcus* species are prevalent, with *Streptococcus salivarius* being a prominent commensal that produces antimicrobial compounds to suppress pathogens and maintain microbial homeostasis (8). *F. nucleatum* acts as a bridge between early- and late-colonizing species (7, 9, 10), forming stable co-aggregates with *Streptococcus gordonii*, thereby promoting *F. nucleatum* (11, 12) growth while modulating host immune responses to prevent excessive inflammation (13, 14). Therefore, illuminating the composition and ecological characteristics of microbes in each body site is of great significance for understanding the relationships between human health and symbiotic microbes.

Currently, identifying microbes closely related to specific human diseases is an important discussion in the study of disease causality (15). Previous studies have mainly relied on statistical methods. For instance, the widely used microbiome-wide association study (MWAS) (16) primarily focuses on identifying characteristic taxa through differential abundance analysis of individual microbes across disease and control groups. However, no microbe exists in isolation; each is intricately linked with others and forms a well-balanced ecological network (17), which creates better living conditions through mutual symbiosis and cooperative metabolism or enables the microbes to evade host immune surveillance (18). These methods ignore the associations among microbes, resulting in a relatively low alignment with actual inter-microbial relationships within the ecological network.

To address this issue, multiple network analysis approaches have been developed for exploring the composition and associations within the microbial ecosystem (19, 20). Microbial differential network methods, such as NetShift (21), NetMoss (22), Microbiome Differential Network Estimation (23), and NetCoMi (24), typically rely on comparative designs, requiring separate case and control groups to infer network perturbations. This limits their applicability in data sets lacking matched controls (25). In contrast, network clustering analysis, which focuses on a single network under specific study conditions, is more suited for scenarios without control groups. Moreover, network clustering simplifies the complexity of the network, enabling the analysis of relationships among a smaller set of groups rather than thousands of taxa (26). Despite the availability of various network clustering tools, such as the Louvain method for community detection (27) and weighted gene co-expression network analysis (WGCNA) (28), there is a lack of clustering algorithms tailored specifically to microbiome data (26), such as the manta clustering algorithm for weighted ecological networks (29). Additionally, integrated microbiome analysis frameworks, such as iNAP (30) and ggClusterNet (31), which combine network construction, differential network analysis, and clustering, have been proposed. However, challenges remain in post-clustering analysis, particularly in identifying biologically meaningful taxon groups and uncovering potential key bacteria within subgroups for biologically relevant inferences (26, 32).

In this paper, we introduce MNetClass, a microbial network clustering framework designed to capture the most relevant information from microbial communities. By accounting for associations between microbes, MNetClass can classify unique subgroups for each location while also exploring the network’s characteristic microbes and their intermicrobial relationships using a Walktrap (33–36) algorithm and rank-sum ratio–entropy weight evaluation model (37, 38). Unlike other methods, MNetClass operates without the need for control samples and directly analyzes microbial features based on their abundance and correlations within the ecological niche. After network partitioning, it effectively identifies central subcommunities, providing deeper insights into microbiome organization and structure. We showcase the capabilities of MNetClass using both simulated and real-world microbiome data sets. Simulation results show that MNetClass outperforms current unsupervised microbial clustering methods. In case studies, application to microbiome data from five distinct oral sites revealed site-specific microbial communities. Additionally, MNetClass demonstrated superior predictive performance on cross-cohort Autism Spectrum Disorder (ASD) data and identified age-related microbial communities across different oral sites, underscoring its wide applicability in microbiome research. MNetClass is implemented in an open-source R package and is freely available at https://github.com/YihuaWWW/MNetClass, providing a novel all-in-one network analysis service for microbiome research.

## MATERIALS AND METHODS

### Overview of MNetClass

In general, our MNetClass framework mainly contains three steps: (i) constructing a microbial association network from a microbial abundance matrix; (ii) partitioning the network into subnetworks using a random walk algorithm; and (iii) evaluating subnetworks and network nodes (microbes) based on topological properties, followed by scoring and ranking them through an integrated rank-sum ratio–entropy weight evaluation model.

For the microbial association network, we utilize the classic Spearman’s correlation method. The microbial correlation network is characterized as a weighted graph G=(V,E,W). In the network, nodes (V) represent bacteria, with the weight of a node denoting the average relative abundance of that bacteria in the ecological niche, and edges (E) represent interbacterial associations, with their weights $\left(W\right)$ being measured by Spearman’s correlation coefficient between microbial taxa. Within a network, communities are subgraphs (or subnetworks) containing more internal connections than external connections, representing microbial subpopulations with strong correlation relationships in the microbial ecosystem (39).

Next, a community partitioning algorithm based on random walks is employed to explore specific, highly interactive microbiota subpopulations within a particular microbial ecosystem. For specificity, scoring models are utilized to compare the topological properties of subnetworks, including network local properties such as degree centrality (40), betweenness centrality (41, 42), closeness centrality (41, 43), and eigenvector centrality (44, 45), as well as global network properties, such as graph diameter (46), modularity (47–49), density (50), and clustering coefficient (51).

To mitigate the influences of magnitude differences among various indicators on the assessment of network topological properties, the integrated rank-sum ratio–entropy weight evaluation model is leveraged to holistically evaluate the composite topological properties of the microbial network in a given niche. The subnetworks or nodes (microbes) with the highest composite score are then selected as the characteristic subgroup and network node bacteria for that particular niche.

### Data pre-processing: normalization and filtration of low-abundance, low-frequency microorganisms

Microbiome data processing begins with a non-negative matrix containing the abundant data of all annotated microbial species in each sample. The data include the total read counts $\omega^{(k）}=[\omega_{1}^{\left(k\right)},\cdots ,\omega_{p}^{k}]$ for p taxa in sample k. Here, $\omega_{i}^{\left(k\right)},i=1,\cdots ,p$ represents the read counts of taxa i in sample k. Due to variations in sequencing depth across samples and the influence of sequencing technologies, the abundance matrix is often sparse, containing a large proportion of zeros. Therefore, we performed preprocessing on the data.

**Fig 1:**
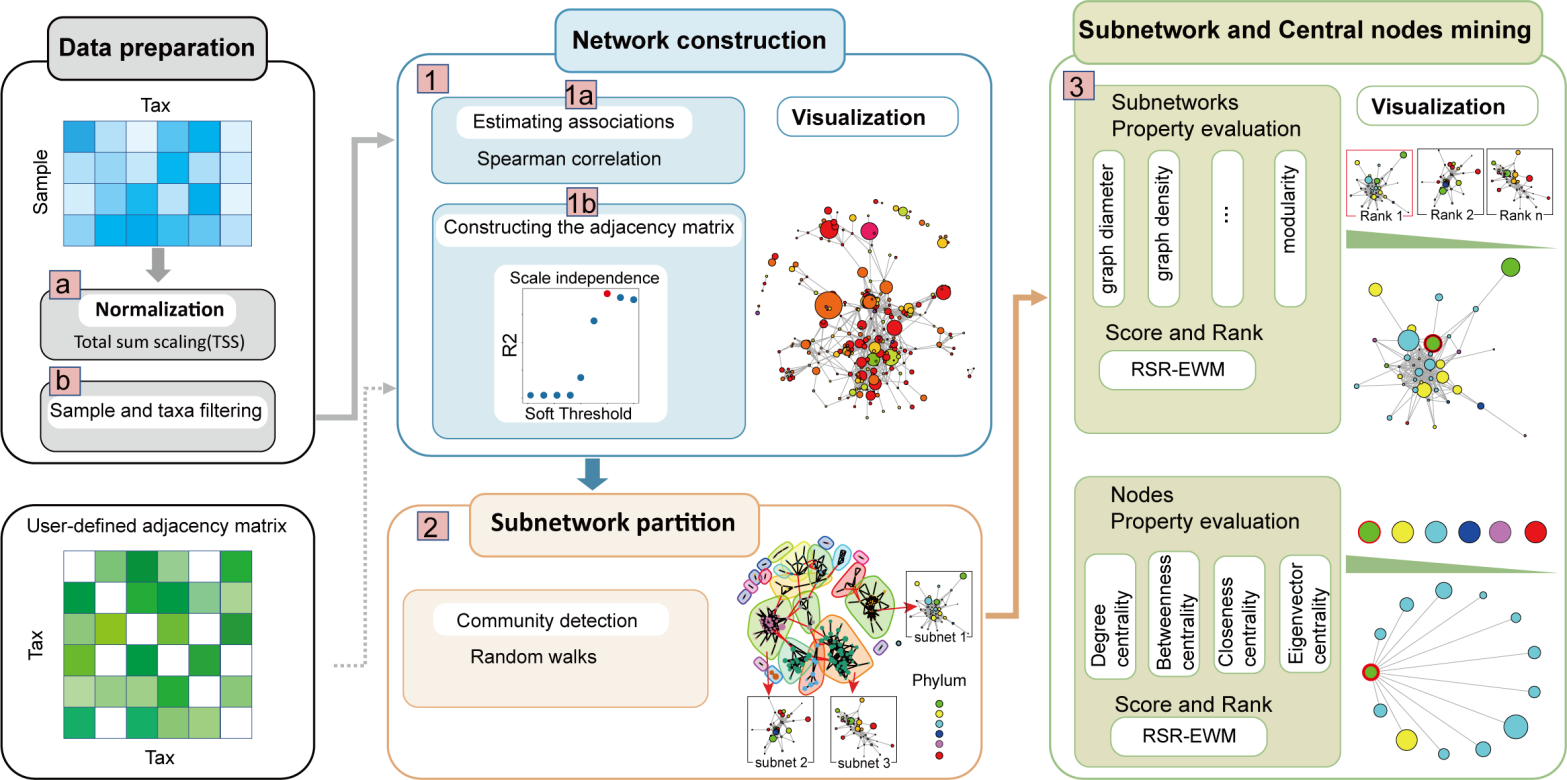
The simplified diagram of MNetClass’s analysis pipeline. Data pre-processing to normalize and filter low-abundance and low-frequency microorganisms. 1. Constructing a microbial association network. 2. Partitioning the network into subnetworks. 3. Evaluating and scoring microbial topological properties of subnetworks and central node microbes.

First, to make the read counts comparable across samples, the microbial sequence counts are normalized into relative abundance using the total sum scaling normalization technique (52, 53) (step a in Fig. 1). Then, the taxa with low abundance or low frequency (step b in Fig. 1) are filtered out to reduce the sparsity of the relative abundance matrix.

### Constructing a microbial association network

#### Correlation analysis among all microbes in a given niche

To ensure the wide applicability of MNetClass, the classical Spearman’s rank correlation coefficient (54, 55) is used to measure correlations between microbial taxa (step 1a in Fig. 1) to obtain the association matrix for microbial correlations in a given niche. Correlation-based techniques remain one of the most popular approaches for analyzing microbial networks in human gut, oral, and soil microbiomes due to their low computational complexity, simplicity, flexibility, and ease of operation (25, 56). These methods have led to significant research discoveries in understanding microbial associations (57–60).

#### Construction of the microbial adjacency matrix

Since the estimated correlations are generally nonzero, directly using them as the adjacency matrix results in a densely connected network. Therefore, the matrix A of the Spearman correlation coefficient is processed through two sparsification steps. First, we introduce a defining threshold and keep the pairs of microbes with absolute correlation coefficients above this threshold (42, 61). Second, Spearman’s rank correlation test (36) is employed to identify correlations that are significantly different from zero. Then, we generate a sparse adjacency matrix $A^{p\times p}$ (step 1b of Fig. 1). In this matrix, the entries $a_{ij}$ represent the correlation coefficients between taxa i and j, and these values serve as the weights for the edges of the microbial network, with the nodes representing the microbial taxa. The diagonal elements, $a_{ii}$, are set to zero, as they represent self-correlations, which are not relevant in this context.

We selected a biologically motivated approach suitable for scale-free networks to determine the optimal correlation coefficient threshold, rather than relying on an entirely arbitrary choice. Scale-free networks exhibit a degree distribution that follows a power-law pattern, characterized by a small number of highly connected nodes (hubs) and a large number of sparsely connected nodes. This distribution has been observed in various biological networks, including microbial co-occurrence networks (28, 60, 62–64). It is also commonly used as a threshold criterion in the widely adopted WGCNA package (65).

To identify the optimal threshold that ensures the resulting network aligns with the scale-free property, we tested a range of correlation thresholds (0–1, in increments of 0.1). For each threshold, the degree distribution of the network was calculated using the igraph package (66). We then fitted the degree distribution to a power-law model using the nonlinear regression function nls in R, based on the frequency distribution of node degrees. The fitted power-law equation was used to predict the response variable (degree frequency) as a function of the predictor variable (degree). The goodness-of-fit was evaluated by calculating $R^{2}$, and the significance of the $R^{2}$ value was tested using a permutation test to obtain the associated *P*-value (67). Finally, the threshold corresponding to the maximum $R^{2}$ value with P≤0.05 was selected as the optimal correlation coefficient threshold.

### Partitioning the network into subnetworks

Previous studies have demonstrated that subnetworks frequently identify functionally coherent microbial groups (68, 69). Community detection methods are instrumental in identifying subgroups within microbial communities that exhibit strong associations. The Walktrap algorithm (35, 36, 70) is employed to partition the sparse weighted network into multiple relatively independent subnetworks. For an undirected network G=(V,E,W) consisting of n=|V| nodes and m=|E| edges, this algorithm can calculate a community structure with a time complexity of O(mnH), where H is the height of the corresponding dendrogram. The primary advantage of this algorithm is that it can produce robust results rapidly without requiring the number and size of the communities as parameters (34). The Walktrap algorithm is based on the general idea that, if one performs random walks on a network, then the walks are more likely to stay within the same cluster due to the assumed higher level of interconnectedness within clusters. Walktrap is slightly slower than the fast greedy approach, but it is shown to be more accurate (35). Other algorithms for community detection can also be used in this method; these algorithms are presented in Supplemental Material.

The measure of similarity between nodes is based on the distance values obtained from the random walk algorithm. Walktrap utilizes certain characteristics of random walks on graphs to define a measure of structural similarity between nodes and communities, thereby defining a distance r (35, 71), as shown in equations 1 and 2. Here, the distance is used in hierarchical clustering algorithms to merge nodes (microbes) into communities.

Definition 1: Let i andj be two nodes in the graph,


(1) $$r_{ij}=\sqrt{\sum_{k=1}^{n}\frac{{\left(P_{ik}^{t}-P_{jk}^{t}\right)}^{2}}{d\left(k\right)}}=\left\|D^{-\frac{1}{2}}P_{i\cdot }^{t}-D^{-\frac{1}{2}}P_{j\cdot }^{t}\right\|,$$

where $\left\|∙\right\|$ denotes the Euclidean norm on $R^{n}$. $r_{ij}$ represents the distance between nodes i and j. Let $P_{ij}^{t}$ represent the probability of a random walk of length t from node i to node j. Furthermore, P denotes the transition matrix of the random walk process. Specifically, P is defined as $P=D^{-1}A$, where A is the adjacency matrix of graph G, and D is the degree matrix ($\forall i,D_{ii}=d(i)\text{and}D_{ij}=0\text{for}i\neq j$).

Definition 2: Let $C_{1},C_{2}\subset V$ be two communities of the graph. We define the distance between the two communities, denoted as $r_{C_{1}C_{2}}$, as


(2) $$r_{C_{1}C_{2}}=\left\|D^{-\frac{1}{2}}P_{C_{1}\cdot }^{t}-D^{-\frac{1}{2}}P_{C_{2}\cdot }^{t}\right\|=\sqrt{\sum_{k=1}^{n}\frac{\;{\left(P_{C_{1}k}^{t}-P_{C_{2}k}^{t}\right)}^{2}}{d\left(k\right)}}.$$

Here, $P_{C_{j}}^{t}\left(P_{C_{j}}^{t}=\frac{1}{\left|C\right|}\sum_{i\in C}P_{ij}^{t}\right)$ represents the probability of reaching node j from community C within t steps. $P_{C∙}^{t}$ is a probability vector. D denotes the degree matrix.

### Evaluating and scoring microbial topological properties of subnetworks and central node microbes

#### Evaluating and scoring microbial subnetworks

In microbial community network analysis, the topological properties of subnetworks can be leveraged to identify key microbial taxa. Studies have indicated that microorganisms with higher topological centrality are more likely to play pivotal roles within the community (59, 72). Therefore, we conducted a topological analysis of the subnetworks derived from community detection, calculating their respective network properties. In each niche, the different subnetworks ranked in the top *k* percentile by node count (*k* = 20) are selected and evaluated by 10 same network topological property indicators, including the graph diameter (46), graph density (50), average path length (46), average degree (73), edge and vertex connectivity (50), clustering coefficient (51), and modularity (47); these are described in Supplemental Material 2.

Next, we identified key subnetworks based on their topological properties. According to previous studies (74–76), selecting subnetworks using multiple topological properties is more reliable than relying on a single property, as the resulting microbial communities are more likely to represent key species. Inspired by this approach, we incorporated all the aforementioned topological properties as selection criteria. However, the scales and relative importance of these properties vary. To address this, an integrated rank-sum ratio–entropy weight (RSR-EWM) evaluation model (38) is used to assess the composite topological properties of each subnetwork. The subnetwork with a topological property score of “1" is chosen to represent the characteristic subgroups in this niche. Since the topological property values used in the process are rank-ordered, representing the relative magnitudes of the data rather than the data itself, there are no special requirements for indicator selection. This makes the rank-sum ratio applicable to various evaluation subjects and capable of identifying minor changes in the objects of study (37). We introduce the entropy weight method to perform a weighted analysis of evaluation indicators (77). The EWM is an objective evaluation method following the principle that the greater the dispersion of an indicator is, the lower its information entropy and the more information it contains.

The specific evaluation process of the RSR-EWM is given below.

##### Compiling the topological property evaluation data into a matrix

Assuming m subnetworks require comprehensive topological property assessment and n evaluative indicators are used, an m by n data matrix can be generated, $X={\left(X_{ij}\right)}_{m\times n}$. Here, $X_{ij}$ represents the numerical value of the jth topological property indicator of the ith subnetwork.

##### Standardizing the subnetwork topological property metrics

For the positive network topological property, where larger values correspond to better network topological properties, the standardization process is expressed as equation 3. For the negative indicators, the standardization process is expressed as equation 4.


(3) $$Z_{ij}=\frac{X_{ij}-min\left(X_{1j},X_{2j},\cdots ,X_{mj}\right)}{max\left(X_{1j},X_{2j},\cdots ,X_{mj}\right)-min\left(X_{1j},X_{2j},\cdots ,X_{mj}\right)},$$

(4) $$Z_{ij}=\frac{max\left(X_{1j},X_{2j},\cdots ,X_{mj}\right)-X_{ij}}{max\left(X_{1j},X_{2j},\cdots ,X_{mj}\right)-min\left(X_{1j},X_{2j},\cdots ,X_{mj}\right)},$$

##### Computing the rank of each indicator

From the m by n data matrix $X={\left(X_{ij}\right)}_{m\times n}$, generated from the m subnetworks and n evaluative indicators, we compile the ranks for each evaluation object for every indicator. The positive network topological property indicators are ranked in ascending order. When identical data are observed for the same indicator, an average rank is assigned, resulting in the rank matrix, R.

##### Weighting of each indicator by EWM

Step 1: Calculation of the proportion, $p_{ij}$, that the ith evaluation object holds for the jth indicator. This proportion, $p_{ij}$, is utilized as the probability in the calculation of relative entropy, as described in equation 5.


(5) $$p_{ij}=Z_{ij}/\sum_{i=1}^{m}Z_{ij}.$$

Step 2: Calculation of the information entropy, $e_{j}$, for each indicator, as detailed in equation 6.


(6) $$e_{j}=-\frac{1}{Inm}\sum_{i=1}^{m}p_{ij}In\left(p_{ij}\right),\;\;j=1,2,3,\cdots ,n.$$

Step 3: Normalization of the information entropy to ascertain the entropy weight, $W_{j}$, of each indicator, as described in equation 7.


(7) $$W_{j}=\left(1-e_{j}\right)/\sum_{j=1}^{n}\left(1-e_{j}\right).$$

##### Computing and ordering the weighted rank-sum ratio

Calculation of the weighted rank-sum ratio (WRSR) as described in equation 8.


(8) $${WRSR}_{i}=\frac{1}{mn}\sum_{j=1}^{n}W_{j}R_{ij}.$$

##### Converting the distribution of WRSR_*i*_ into probability units

The ${WRSR}_{i}$ distribution is represented by the cumulative frequency in the probit unit table.

Step 1: Arrange the values of ${WRSR}_{i}$ in ascending order.

Step 2: List the frequency f of each group and calculate the cumulative frequency ∑f.

Step 3: Determine the average rank $\overset{-}{R_{i}}$ of each evaluation object. For ${WRSR}_{i}$ with a frequency equal to 1, the value of $\overset{-}{R_{i}}$ is the rank of ${WRSR}_{i}$. For ${WRSR}_{i}$ with a frequency not equal to 1, the value of $\overset{-}{R_{i}}$ is the average of each rank.

Step 4: Calculate the downward cumulative frequency as $\overset{-}{R}/m\times 100\%$ and correct the final item with $\left(1-1/4m\right)\times 100\%$.

Step 5: Convert the downward cumulative frequency to the Probit probability unit. The Probit is the standard normal deviation *µ* plus five corresponding to the cumulative frequencies, as referenced in the “comparison table of percentages and probability units” (78).

##### Computing the value of WRSR

Taking the Probit probability unit corresponding to the cumulative frequency as the independent variable and ${WRSR}_{i}$ as the dependent variable, we compute the linear regression equation as depicted in equation 9. This regression equation is then subjected to rigorous statistical testing.


(9) $$WR\hat{S}R=a+b\times Probit.$$

##### Ranking and binning

In accordance with the probabilistic unit Probit and the adjusted rank-sum ratio value $WR\hat{S}R$, binning is conducted.

Based on the scores derived from the RSR-EWM model, the top-scoring subnetworks are selected for each respective group in the study.

### Evaluating and scoring central node microbes

Furthermore, within the selected subnetworks, we identified nodes with favorable composite topological properties, which we refer to as “central node microbes.” This approach is analogous to selecting hub microbes, as described in previous studies, which are characterized by having the highest degree of connectivity (79). These central node microbes are likely to play significant physiological roles within the selected subnetworks (74).

The pipeline of central node microbes is similar to that of subnetwork analysis. We assess the topological attributes of the chosen subnetwork nodes (microbes) using five network centrality measures: degree (41), weighted degree (41), betweenness (41, 42), closeness (41), and eigenvector centrality (44, 45), as provided in Supplemental Material 2. Next, a node’s composite score is determined using an RSR-EWM evaluation model. Nodes highly ranked by composite topological property indicators are selected as the core microbes within this characteristic subgroup (80).

### Implementation of the MNetClass as an R package

The MNetClass methodology has been implemented as an R package for assisting easy analysis of microbial association networks obtained from distinct body sites of human microbiome studies available at https://github.com/YihuaWWW/MNetClass. The input data for this R package consist of two components: a microbial abundance and the corresponding “species-phylum” or “genus-phylum” relationship. MNetClass comprises three functions: “comm,” “netscore,” and “nodescore.” The “comm” function enables the construction of correlation networks, partition of networks, calculation of network and node topological properties, as well as visualization of association networks and network partitioning results. The “netscore” and “nodescore” functions, respectively, facilitate the comprehensive scoring and ranking of subnetworks and node topological properties using RSR-EWM.

### Data sets

#### 16S rRNA sequencing data from distinct oral sites

We collected samples from the gingival crevicular fluid (GCF), dental plaque (P), buccal mucosa (B), saliva (S), and tongue coating (T) of 21 volunteers, and all samples were analyzed by 16S rRNA sequencing. After primary sequencing data quality control and clean read clustering, we annotated the operational taxonomic units by the Ribosomal Database Project (RDP version 2.14 [see reference 81]) and obtained a total of 810 genera and 37 phyla, as described in Supplemental Material 3 and Table S1.

#### Synthetic data sets

We utilized a modified version of the FABIA R package (82) to simulate microbial abundance matrices, which is designed for generating synthetic data to evaluate biclustering algorithms. The makeFabiaDatablocksPos function was adapted to generate biclusters at predefined locations, which were designated as true-positive clusters. The simulation was repeated 50 times to produce 50 sets of synthetic microbial abundance data. The constructed matrices were subsequently used to infer correlation networks.

#### Gut microbiome data across Autism Spectrum Disorder cohorts

In this study, we analyzed gut microbiome data and metadata from individuals with Autism Spectrum Disorder sourced from publicly available data sets described by Xu et al. (83). The data comprise 1,019 samples (569 ASD and 450 controls) from 10 cohorts, with detailed metadata including age, country, sequencing region, and platform. Sequencing was performed using 16S rRNA gene sequencing, and taxonomic profiling was conducted using the RDP taxonomy outline (version 14). To ensure comparability across cohorts, sequences were normalized to relative abundances. This data set provides a robust resource for studying gut microbial patterns in ASD and enables cross-cohort comparisons to assess the consistency of microbial signatures.

#### Oral microbiome data stratified by age

In this study, we utilized oral microbiome data sets from the work of Liu et al. (84), which examined microbial community changes across five age groups (age 11–15, 18–20, 28–32, 38–45, and 50–65) and three oral sites of healthy people (gingival crevicular fluid, saliva [SAL], and tongue back [TB]). The data set includes 179 samples, with detailed metadata describing participant age, gender, and sampling sites. Sequencing was performed using 16S rRNA gene sequencing on the Illumina MiSeq platform, targeting the V3–V4 region. The original study revealed significant shifts in microbial composition both with increasing age and across different oral sites. Our study leveraged this data set to show the real-world applicability of MNetClass and performed case analyses.

## RESULTS

### Application of the MNetClass framework on 16S rRNA sequencing data from distinct oral sites

To test the efficacy of MNetClass, we implemented our pipeline on collected samples from the GCF, P, B, S, and T of 21 volunteers.

#### Data pre-processing: data filtering, normalization, and zero handling

A total of 282, 322, 339, 241, and 225 genera were obtained in the oral site GCF, P, B, S, and T, respectively. We normalized the microbial genus counts into relative abundance at each oral site and filtered out the genus with a relative abundance < 0.0002 or an occurrence <30%.

#### Construction of microbial association networks

**Fig 2:**
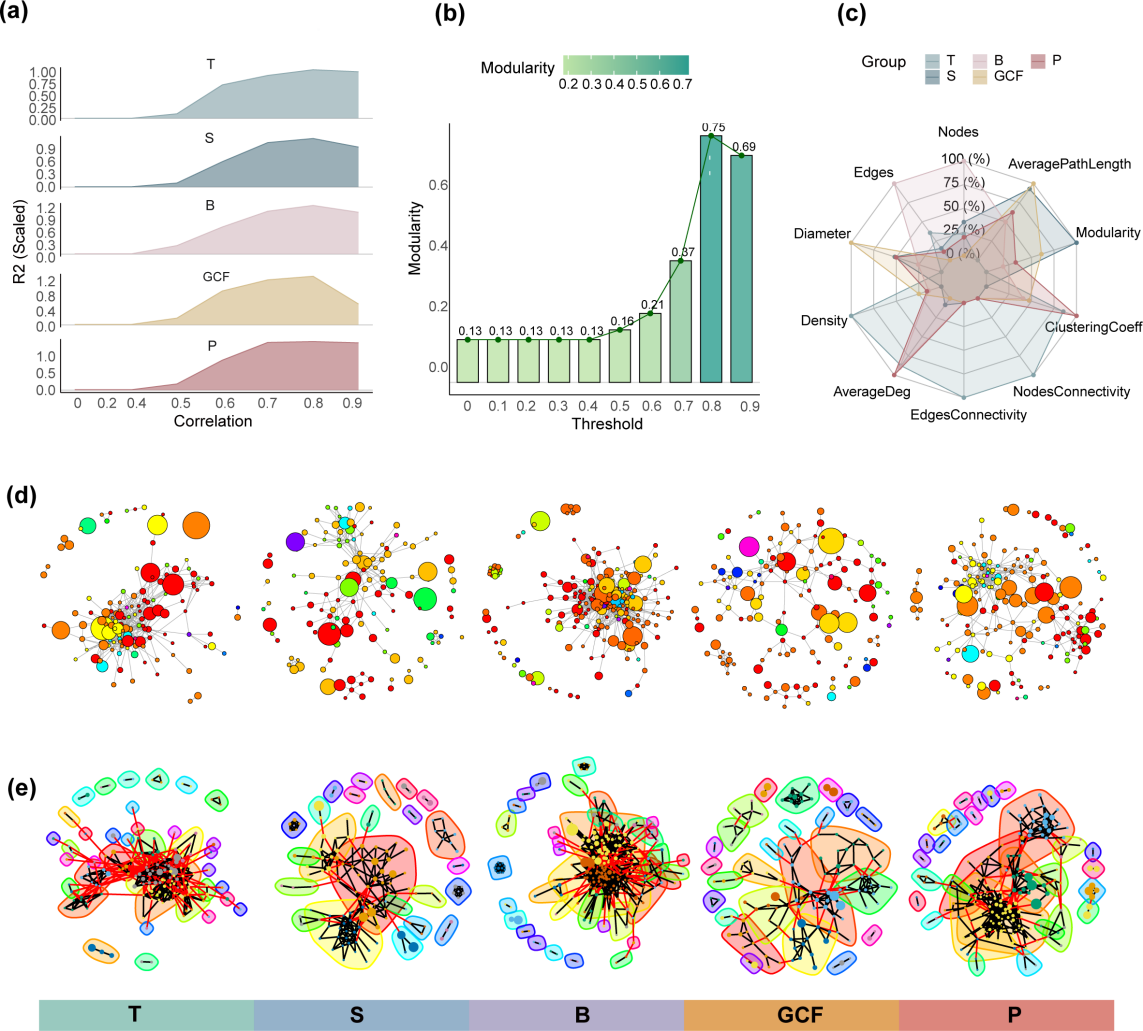
Construction and partitioning of correlation networks. (**a**) Distribution of $R^{2}$ values for the scale-free network fit at different correlation coefficient thresholds. Different colors represent five oral sites: gingival crevicular fluid, dental plaque, buccal mucosa, saliva, and tongue coating. (**b**) Modularity of subnetworks after partitioning based on Walktrap at different Spearman coefficient thresholds. (**c**) Radar plot of topological properties of subnetworks identified from five oral sites. (**d**) Visualization of correlation networks constructed for the five oral sites. Nodes represent bacteria, with node size corresponding to relative bacterial abundance. Node color indicates distinct phylum, and edges between nodes signify correlations between bacteria. (**e**) Visualization of the partitioned subnetworks. Node color represents the bacteria belonging to different clusters. Black edges indicate positively weighted correlations, signifying positive correlations between the connected bacteria, while red edges represent negatively weighted correlations, indicating negative correlations.

For the selection of the Spearman correlation coefficient threshold, we constructed microbial association networks for five different oral sites at various thresholds and calculated the $R^{2}$ and *P*-values for the fit to a scale-free network (Fig. 2a). We found that, for all five sites, the network constructed with a threshold of 0.8 yielded the highest $R^{2}$, indicating the best fit to a scale-free network. Therefore, we set the Spearman correlation coefficient threshold to 0.8 for network construction using the microbial data sets from these oral sites.

Additionally, using the saliva data as an example, we tested the modularity of subnetworks formed at different correlation coefficient thresholds (Fig. 2b). We observed that a threshold of 0.8 resulted in higher modularity, facilitating better network partitioning.

Spearman correlation coefficients were applied and filtered at each oral site with a correlation coefficient > 0.8 and a corrected *P* value < 0.05. The retained pairs of genera were used to construct the adjacency matrices. For each oral site, “GCF,” “P,” “B,” “S,” and “T” obtained 223, 466, 1,128, 246, and 558 pairs of correlated genera, respectively, as described in Table S2.

#### Variation of network partition in five oral sites

The correlation networks for each oral site were constructed based on the adjacency matrices (Fig. 2d). Subnetwork partitioning was then performed using the Walktrap community partition algorithm, with the random walk step length set to four. The partitioning networks of five oral sites are shown in Fig. 2e and Table S3. Specifically, “GCF” is divided into 25 subnetworks, with six encompassing more than 10 nodes (Table S3-1). “P” is partitioned into 29 subnetworks, with four encompassing more than 10 nodes (Table S3-2). “B” is partitioned into 35 subnetworks, with three encompassing more than 10 nodes (Table S3-3). “S” is divided into 24 subnetworks, with three encompassing more than 10 nodes (Tables S3-4). “T” is partitioned into 37 subnetworks, with two encompassing more than 10 nodes (Table S3-5).

#### Identification of subnetworks and central node microbes

**Fig 3:**
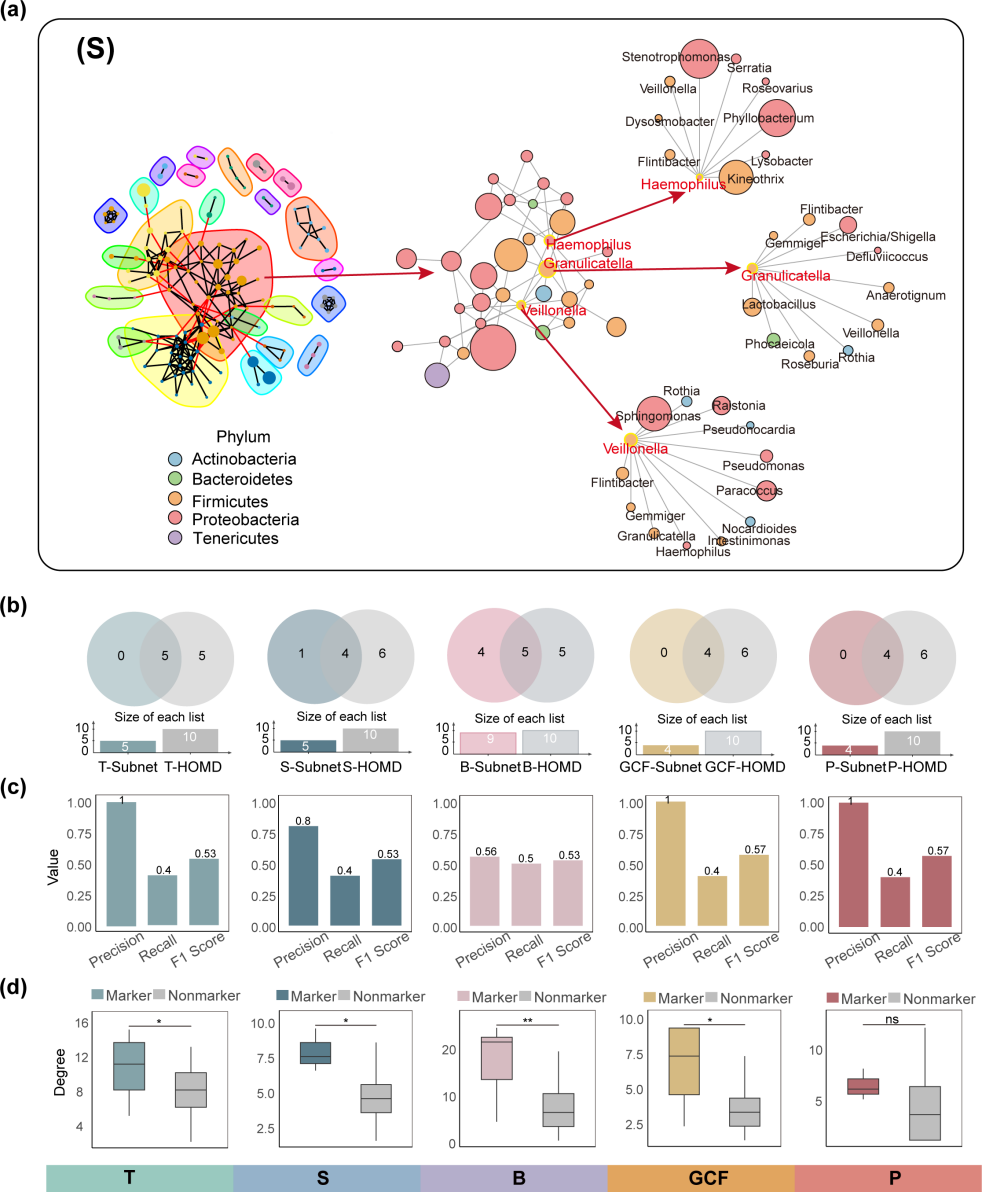
Identification and evaluation of key subnetworks and central microbes. (**a**) Key subnetworks and central microbes in the saliva identified using the Walktrap algorithm and the integrated rank-sum ratio–entropy weight evaluation model. The left panel shows a visualization of the subnetwork partitioning results. The middle panel highlights the important subnetworks selected from the left panel. The right panel displays the key microbial nodes and their associated bacteria selected from the middle panel. Red labels denote key microbial nodes, while black labels indicate bacteria associated with them. (**b**) Venn diagram of phyla in subnetworks identified by MNetClass from five oral sites and core phyla from different sites in the mHOMD database. (**c**) Barplot showing the performance of MNetClass on mHOMD benchmarks, evaluated using Precision, Recall, and F1-score. (**d**) Connection strength of each node in subnetworks identified by MNetClass from five oral sites. Red represents microbial markers in the mHOMD database, and gray represents other bacteria. In the box plots: center line, median; box, IQR (the range between the 25th and 75th percentiles); whiskers, 1.5× IQR; dots, outliers. Two-sided Wilcoxon test. ** *P ≤* 0.01, * *P ≤* 0.05; ns > 0.05.

For each oral site, the top 20% of subnetworks (*k* = 20) in terms of node count were selected and evaluated by 10 kinds of network topological property indicators, which are shown in Table S4. Subsequently, an integrated rank-sum ratio–entropy weight model was applied to assess the overall topological characteristics of each subnetwork. The subnetwork with the highest composite score was designated as the dominant subgroup for each site (Fig. 3a; Fig. S1 to S4). Notably, the topological properties of the selected subnetworks varied across different oral sites (Fig. 2c). For instance, in the S group, the dominant subnetwork comprised 32 nodes and 68 edges, with a graph diameter of 4.194 and a graph density of 0.137, among other metrics (Table 1). The corresponding topological details for other sites are provided in Supplemental Material 5. In a similar manner, we computed five centrality-based topological properties for all nodes within the selected subnetworks. Using the RSR-EWM model, we ranked the nodes according to their integrated centrality scores and identified the highest-ranking taxa as the central microbes of each subnetwork. In the S group, *Veillonella*, *Granulicatella*, and *Haemophilus* emerged as the top three central taxa, with their centrality metrics shown in Table 2. For the other oral sites, the central taxa identified were as follows: *Streptococcus* and *Neisseria* in the GCF group; *Abiotrophia*, *Lachnospira*, and *Porphyromonas* in the P group; *Actinomyces*, *Veillonella*, and *Campylobacter* in the B group; and *Rothia*, *Prevotella*, and *Gemella* in the T group. Detailed topological metrics and evaluation procedures for all subnetworks and central microbes across the five oral sites are provided in Supplemental Material 5.

**TABLE 1:** Topological properties of selected subnetworks

Subnetwork ID	Node number	Edge number	Graph diameter	Graph density	Average degree	Edge connectivity	Node connectivity	Clustering coefficient	Modularity	Average path length
1	32	68	4.194	0.137	4.250	1	1	0.331	0.466	2.185
5	24	67	4.137	0.243	5.583	1	1	0.472	0.237	1.747
4	12	19	4.077	0.288	3.167	1	1	0.476	0.263	1.772
2	9	11	3.323	0.306	2.444	1	1	0.250	0.252	1.690
16	5	10	0.969	1.000	4.000	4	4	1.000	−0.080	0.924

**TABLE 2:** Topological properties of selected nodes in subnetwork 1

Nodes	Degree centrality	Weighted degree centrality	Betweenness centrality	Closeness centrality	Eigenvector centrality	RSR	Rank
Veillonella	9	7.480	151	0.021	1.000	4.778	1
Granulicatella	8	6.692	73	0.018	0.744	4.391	2
Haemophilus	7	5.835	88	0.019	0.578	4.287	3

#### The microbial communities identified by MNetClass have high biological relevance to five oral sites

To enhance the credibility and interpretability of the experimental results, we will elucidate the reliability and biological significance of the key microbial subnetworks identified by the MNetClass framework.

##### Reliability evaluation of microbial community identification

We evaluated the reliability of the MNetClass framework in identifying key microbial communities across different oral sites, based on previous studies (85). As a reference standard, we utilized the expanded Human Oral Microbiome Database (eHOMD) (86), which offers a comprehensive profile of microbial distributions across distinct oral sites, including curated core taxa specific to each niche. To assess concordance, we compared the phyla of key microbial communities identified by MNetClass across five oral sites with the core phyla included in eHOMD (Fig. 3b) and computed standard performance metrics, including precision, recall, and F1-score (Fig. 3c). We found that all genera comprising the key microbial community in the tongue coating were affiliated with phyla present in eHOMD. For the saliva, 80% of genera belonged to eHOMD core phyla, while for the buccal mucosa, this proportion was 56%. In contrast, both the gingival crevicular fluid and plaque exhibited complete phylum-level overlap (100%) with eHOMD core taxa. These results indicate that the majority of key microbial taxa identified by MNetClass correspond to the eHOMD-defined core phyla, underscoring the framework’s accuracy in detecting ecologically relevant taxa.

We further categorized genera identified by MNetClass as marker bacteria if they were present in the eHOMD core taxa and as non-marker bacteria otherwise. Notably, the connection strength of marker bacteria was significantly higher than that of non-marker bacteria in the microbial networks of the tongue, saliva, buccal mucosa, and GCF (Fig. 3d). Although a similar trend was observed for the dental plaque (P), the difference was not statistically significant based on the Wilcoxon test. These findings highlight the crucial role of marker bacteria in shaping site-specific microbial networks within the oral cavity.

##### Biological significance of identified central bacteria

To explore the biological relevance of the identified central bacteria, we focused on group S as an example. In this group, MNetClass identified *Veillonella*, *Granulicatella*, and *Haemophilus* as central taxa. Notably, *Veillonella* and *Haemophilus* are among the 10 most abundant genera in saliva according to the eHOMD database. Moreover, their phylum, *Bacillota* (formerly *Firmicutes*), is likewise among the 10 most abundant salivary phyla. These findings suggest that the majority of central taxa identified in our analysis belong to the core microbiota of this ecological niche.

Theoretically, central taxa possess high topological centrality and occupy key positions within microbial networks, implying a potential for broad ecological influence. In the subnetwork we identified, *Granulicatella* was connected to both *Veillonella* and *Lactobacillus*, reflecting significant co-abundance relationships. Previous studies have reported that *Granulicatella*, together with *Veillonella* and *Streptococcus*, relies on salivary glycoproteins and their degradation products and collaborates through mechanisms such as metabolic cooperation to maintain oral microbial homeostasis (87–89). Additionally, in our selected subnetwork, *Haemophilus* was also linked to *Veillonella*, suggesting a significant correlation in their abundance. Although the molecular mechanisms underpinning *Haemophilus-Veillonella* interactions in saliva remain unclear, both genera are known to co-aggregate with taxa such as *Streptococcus* and *Actinomyces*, facilitating co-localization within shared biofilm environments (90, 91). Such spatial proximity may support indirect interactions through metabolic complementarity or signal exchange. Collectively, these observations indicate that the central taxa identified by MNetClass are not only topologically important within the network but also more likely to engage in ecological interactions with other community members. Their presence highlights potential physiological roles within their niche and provides a valuable reference point for future mechanistic investigations.

### MNetClass equals or outperforms other algorithms on synthetic data sets

To evaluate the performance of MNetClass relative to other methods, we generated 50 synthetic data sets using FABIA (82). Originally designed for evaluating biclustering of gene expression data, FABIA was selected for its ability to provide true labels, which serve as ground truth for performance assessment. Furthermore, it has been widely used in previous microbial network analysis studies (29) to generate synthetic data sets for algorithm evaluation.

The accuracy of the identified microbial clusters was assessed using three metrics: complex-wise sensitivity (Sn), cluster-wise positive predictive value (PPV), and geometrical accuracy (Acc) (92). Sn represents the proportion of correctly identified positive samples, reflecting how many of the true positives were accurately detected. PPV indicates the proportion of identified microbial clusters that are true positives. Acc, which combines Sn and PPV using their geometric mean, provides a balanced evaluation of both metrics. We compared MNetClass with five other community detection algorithms: edge betweenness, label propagation, leading eigenvector, fast greedy, and Louvain, as well as the biological weighted network clustering algorithm manta and weighted gene co-expression network analysis using these evaluation metrics.

**Fig 4:**
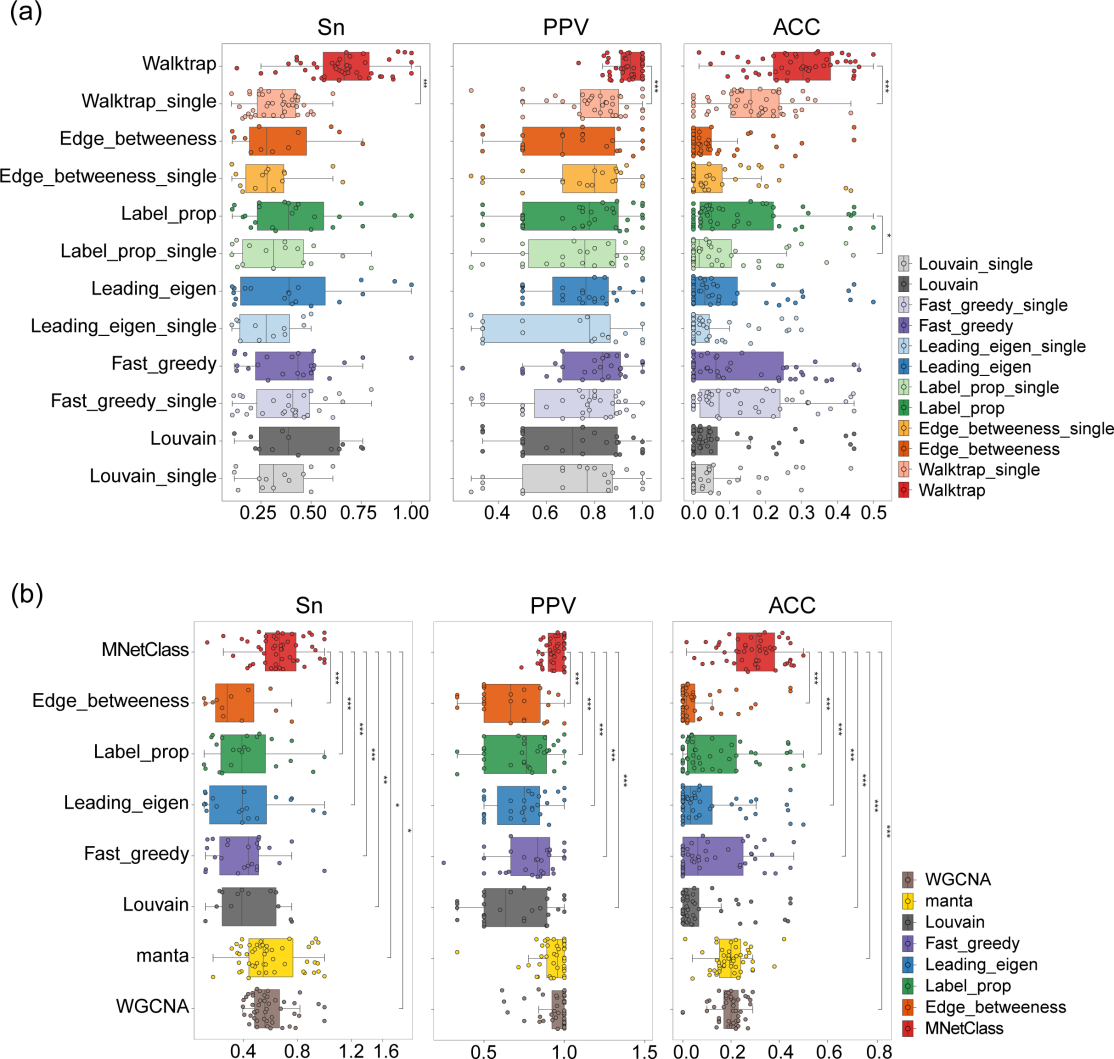
Performance of network clustering tools on 50 synthetic data sets generated with FABIA. (**a**) Evaluation of subnetwork selection using six methods, comparing the performance of individual topological properties versus the RSR-EWM scoring method, based on Sn, PPV, and Acc. Sensitivity, PPV, accuracy, and separation (Sep) were calculated as previously described. “Single” in the legend denotes the use of a single topological property scoring method, while the absence of “single” indicates the use of the RSR-EWM scoring method by default. (**b**) Comparison of the microbial clusters identified by MNetClass and seven other algorithms, based on Sn, PPV, and ACC.

After partitioning the subnetworks using community detection methods, the most likely subnetworks to perform physiological functions were identified based on network topology analysis (74). Previous studies typically used a single optimal topological property to extract subnetworks (93, 94); however, different topological properties yield different optimal subnetworks. As noted in recent literature, selecting subnetworks based on multiple topological properties is a more reasonable approach (95). Therefore, we developed an integrated RSR-EWM evaluation model to provide a comprehensive score for network topological properties. We compared the performance of various community detection algorithms and the subnetworks selected based on single topological properties versus those selected using the RSR-EWM score across 50 synthetic data sets (Fig. 4a). Except for the edge betweenness algorithm, where the subnetwork selected using a single topological property showed a higher ACC score than the RSR-EWM method, with no significant difference, all other algorithms performed better or equally well when combined with the RSR-EWM scoring. Notably, the Walktrap algorithm, when coupled with RSR-EWM, significantly outperformed the single topological property method across all three metrics. Furthermore, when generating box plots for these metrics, we excluded outliers and observed that MNetClass retained the highest number of points, indicating its stability and robustness across the 50 synthetic data sets.

We compared MNetClass with seven other similar algorithms across 50 synthetic microbial data sets. For the microbial network construction step, edge betweenness, label propagation, leading eigenvector, fast greedy, Louvain, and the biological weighted network clustering algorithm manta did not specify a particular method for network construction, so we used the same approach as MNetClass. After network clustering, edge betweenness, label propagation, leading eigenvector, fast greedy, and Louvain selected key subnetworks based on the RSR-EWM topology scoring method, while manta selected the cluster with the highest proportion of strong nodes from the clustered groups as the key subnetwork. For WGCNA, originally designed for gene expression data, we adapted it for microbial co-occurrence network construction and module identification. We used default parameters to automatically construct the network and identify the modules most strongly associated with phenotypes as the key subnetworks. The identified key subnetwork nodes were treated as predicted positives, and the Sn, PPV, and ACC were calculated by comparing these predicted positives with the true labels, as shown in Fig. 3b. MNetClass significantly outperformed the other six algorithms in Sn and ACC and was significantly better than five other community detection algorithms in PPV, with no significant difference when compared to manta and WGCNA.

### Performance evaluation of MNetClass for identifying microbial key subnetwork and ASD prediction

#### Identifying microbial key subnetworks across multiple cohorts

**Fig 5:**
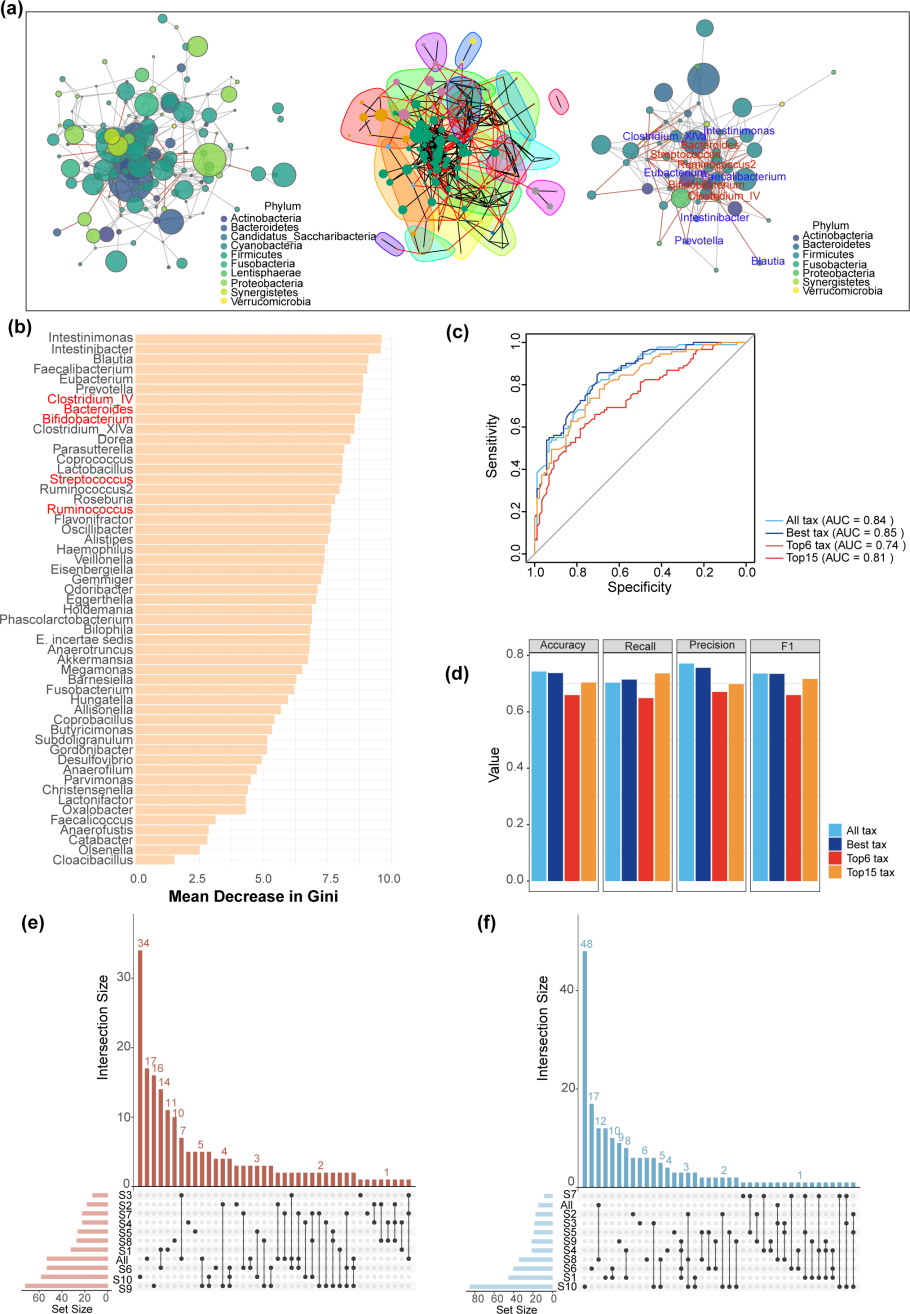
Benchmarking ASD prediction across multiple cohorts. (**a**) Visualization of the network construction, subnetwork partitioning, and subnetwork selection processes for ASD data sets using MNetClass. In the right panel, nodes with blue labels represent the top 10 genera ranked by importance in the random forest model shown in panel **b**, while nodes with red labels represent genera previously validated as ASD-associated in the literature. (**b**) Importance ranking of microbial genera identified by MNetClass in the S1ASD cohort, based on the random forest model. (**c**) Receiver operating characteristic curves for ASD prediction using the random forest model on cohorts S2 to S10. (**d**) Bar plots comparing the accuracy, recall, precision, and F1-score of random forest models constructed using microbial communities identified by MNetClass. (**e and f**) UpSet plots showing the microbial communities identified by MNetClass in the ASD and Healthy group across 10 cohorts.

We applied our algorithm, MNetClass, to identify key microbial subnetworks across 10 independent ASD cohorts, following the workflow outlined in Fig. 1. The identified microbial clusters are visualized in Fig. 5a. Compared to the original method, MNetClass revealed higher overlap in microbial clusters between the ASD and Control groups across all 10 cohorts, indicating greater consistency and reproducibility (Fig. 5e and f). These results suggest that MNetClass effectively captures robust and shared microbial community structures.

#### Predictive performance using identified subnetworks

To evaluate the predictive utility of the identified microbial key subnetworks, we utilized Project S1 as the training set and applied fivefold cross-validation to construct a random forest model. Feature selection was performed using recursive feature elimination to determine the optimal number of features. Subsequently, we validated the model using external data sets, testing four configurations of features derived from the identified subnetworks: all taxa, the optimal number of taxa, and the top 6 and top 15 taxa ranked by feature importance (Fig. 5b).

The ROC curves for these configurations are shown in Fig. 5c. Notably, using the optimal number of taxa, all taxa, and the top 15 taxa yielded superior predictive performance compared to the original method, with AUC scores of 0.85, 0.84, and 0.81, respectively. Other four evaluation metrics were used: accuracy, recall, precision, and F1-score (Fig. 5d). These results underscore the effectiveness of MNetClass in identifying predictive features and constructing robust classification models.

### Case study of MNetClass on oral microbial communities across age groups

#### Subnetwork-related phenotypes as main drivers of sample grouping in oral microbiome data

**Fig 6:**
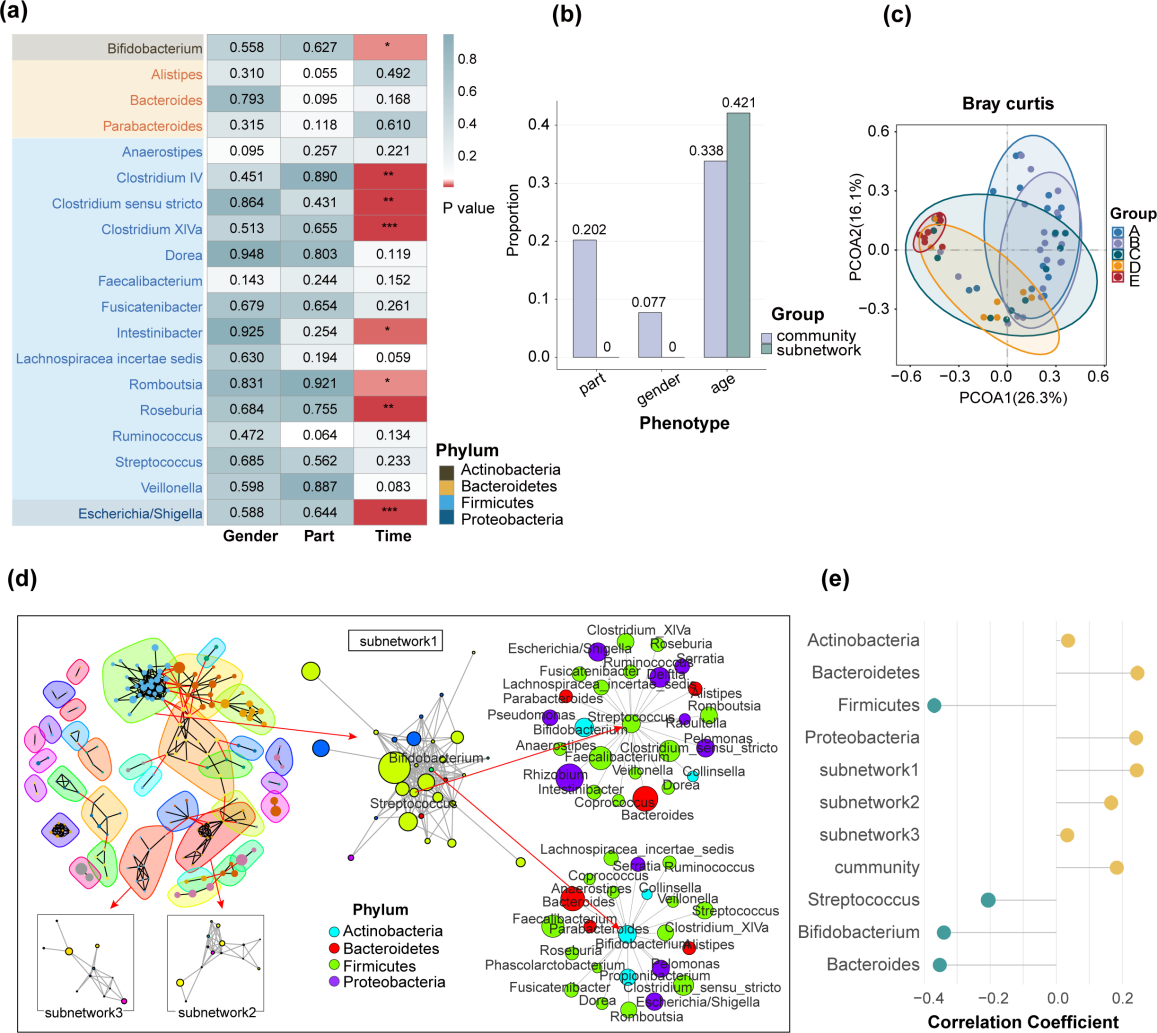
Application of MNetClass for identifying age-associated oral bacteria. (**a**) Heatmap of *P*-values from ANOVA comparing genus in the key subnetworks identified by MNetClass across different phenotypes. In the heatmap, P≤0.05 is marked with “*,” P≤0.01 with “**,” P≤0.001 with “***,” and P≥0.05 with the corresponding *P*-value. (**b**) Proportions of genus in the overall microbiome and those identified by MNetClass in the subnetworks that show significant correlation with different phenotypes, based on ANOVA. Genus with *P* ≤ 0.05 is considered significantly correlated. (**c**) Principal coordinate analysis (PCoA) of Bray-Curtis dissimilarities for sample compositions, with different colors representing different age groups: A (age 11–15), B (age 18–20), C (age 28–32), D (age 38–45), and E (age 50–65). (**d**) Visualization of network analysis results for GCF samples using MNetClass. Subnetworks 1–3 are ranked by the RSR-EWM scoring model, corresponding to rank-1, rank-2, and rank-3 subnetworks. (**e**) Average Spearman correlation coefficients between microbial relative abundances and age. “Subnetwork 1-3” represents the genus within each subnetwork, while “Community” refers to the overall microbiome. Phyla identified by MNetClass, including *Actinobacteria, Bacteroidetes, Firmicutes,* and *Proteobacteria,* are also shown along with the genus they contain.

We ran MNetClass on genus-level oral microbiome data from all samples and performed a correlation analysis between the relative abundance of microbes in the identified key subnetwork and various phenotypes. Since the phenotypes—part, gender, and age group—are categorical variables, we conducted one-way ANOVA to assess whether the relative abundances of microbes significantly differed across categories (*P* ≤ 0.05). The results are shown in Fig. 6a and Table S6.

The analysis revealed that the proportions of microbes with significant abundance differences across the phenotypes part, gender, and age in the identified subnetwork were 0, 0, and 0.421, respectively (Fig. 6b). Additionally, we performed ANOVA on the relative abundance of all microbes with respect to the phenotypes, as detailed in Table S7. Here, the proportions of microbes with significant differences across the phenotypes were 0.202, 0.077, and 0.338 (Fig. 6b).

Our findings indicate that the subnetwork identified by MNetClass is more strongly correlated with the phenotype “age.” This is consistent with the original study, which showed that “part” and “age” were the major drivers of sample classification, as indicated by permutational multivariate analysis of variance (PERMANOVA). We used PERMANOVA based on Bray-Curtis dissimilarities to test differences in full microbial community composition across phenotypes. The *P*-values for “part,” “gender,” and “age” were 0.001, 0.056, and 0.023, respectively. We then constructed a new matrix using only the taxa identified in the subnetwork by MNetClass and repeated the PERMANOVA using the same dissimilarity measure. The *P*-values for “part,” “gender,” and “age” were 0.001, 0.654, and 0.001, respectively. These results demonstrate that our identified subnetwork enhances the significance of “age” as a factor influencing groupings, while reducing the influence of “gender” to a statistically insignificant level. This confirms the need to study age-related oral microbiomes by separate anatomical sites, as suggested by the original analysis.

#### MNetClass identifies age-related microbial communities across different oral sites

We used the GCF samples as an example for principal coordinates analysis, as shown in Fig. 6c. A PERMANOVA test was performed to assess the influence of age on the sample grouping, with a significant *P*-value of 0.001. We then applied MNetClass to identify subnetworks (Fig. 6d). Spearman correlation analysis was conducted between age and each genus in the rank-1, rank-2, and rank-3 subnetworks, as well as in the overall microbial communities. The average correlation values are shown in Fig. 6e. The rank-1 subnetwork exhibited the highest average absolute Spearman correlation coefficient with age (0.2435, 0.1661, 0.0271, and 0.1773), indicating that the key subnetwork we selected is highly correlated with age.

The phyla of the microbes identified in the key subnetworks by MNetClass include *Actinobacteriota* (formerly *Actinobacteria*)*, Bacteroidota* (formerly *Bacteroidetes*)*, Bacillota* (formerly *Firmicutes*)*,* and *Proteobacteriota* (formerly *Proteobacteria*). In comparison, the phyla identified in the original study (84) were *Actinobacteria, Bacteroidetes, Firmicutes, Proteobacteria, and Spirochaetes* (formerly *Spirochaetaeota*)*,* with only one phylum not overlapping. This suggests that the microbial communities identified by MNetClass are consistent with those found in the original study.

Furthermore, we performed a topological analysis of the microbes in the identified subnetwork. Using RSR-EWM, we calculated the integrated topological scores of the nodes and selected those with the highest scores as the central taxa in the subnetwork. These microbes are more likely to play a physiological role in these subcommunities. Notably, the central genera identified in the key subnetwork by MNetClass, *Streptococcus* and *Bifidobacterium*, were also age-related taxa found in the original study (84), supporting the biological relevance of our findings. Theoretically, central taxa with high topological centrality occupy pivotal positions within microbial networks and are more likely to influence the structure and function of the community. *Streptococcus* is a dominant and functionally important genus in the healthy oral microbiome. Several species within this genus, including *Streptococcus mitis* (96) and *Streptococcus sanguinis* (97, 98), are age-associated, exhibiting higher abundance during childhood and a gradual decline with aging. It contributes to maintaining oral microbial homeostasis by co-colonizing with other taxa and promoting community stability (99). In the subnetworks we identified, *Streptococcus* showed significant co-abundance correlations with *Veillonella* and *Bacteroides*, both of which have been previously reported to engage in metabolic cooperation with *Streptococcus* (100, 101). Specifically, *Streptococcus* ferments carbohydrates to produce lactate, which serves as a substrate for lactate-utilizing microbes such as *Veillonella* (100, 102). *Bacteroides*, often considered late colonizers, utilize polysaccharide or protein degradation products, contributing to a complementary metabolic network (101, 103). In addition, *Streptococcus* is recognized as an early colonizer in oral biofilms, facilitating the adhesion and colonization of subsequent microbial species (104). It frequently forms co-aggregates with other taxa (105, 106) and plays a critical role in maintaining oral microbial health through ecological interactions, metabolic networking, and immune modulation (107). While *Bifidobacterium* is typically more abundant in the gut, it has also been detected in gingival crevicular fluid, dental plaque, and saliva. It may confer oral health benefits through acid production, resource competition, and interference with biofilm formation (108). In our subnetworks, *Bifidobacterium* was also correlated with *Streptococcus*, suggesting potential ecological interaction. Previous studies have demonstrated that *Bifidobacterium* inhibits cariogenic pathogens, such as *Streptococcus mutans*, through acidification and competitive exclusion, thereby promoting oral microbial balance (109, 110).

## DISCUSSION

Currently, most microbiome studies typically rely on statistical methods, such as the MWAS (16), and are not based on the principle of ecological theory (111). Microbial network-based methods, such as NetShift (21), NetMoss (38), and manta (29), are thus becoming powerful tools for elucidating the characteristics of microbiome ecosystems. However, most of these methods require a control group and are only suitable for measuring changes in microbial networks under healthy and diseased states. Moreover, very few methods exist for identifying subcommunities after clustering across ecological niches and defining the microorganisms that play an important role in these subpopulations. To address this issue, we established a control-free microbial network clustering analysis framework, MNetClass, which expands the research methods on microbial interrelationships and ecological characteristics.

In MNetClass, we used the random walk community partition algorithm (35) to divide the constructed association network into different subnetworks. This method is appropriate for the microbial network analysis constructed under different habitats because it does not require providing the number and size of partitions as parameters (36). Additionally, the random walk algorithm can yield satisfactory results within a relatively small time complexity, ensuring the efficiency of our overall network analysis (70). However, in our current implementation, the random walk-based model assumes non-negative edge weights and thus cannot directly accommodate negative correlations. To address this, we convert negative correlation values to their absolute values before incorporating them into the network. We acknowledge that this approach has limitations, as negative correlations may sometimes complicate biological interpretation and, in some cases, ignoring negative correlations could be preferable. However, our additional analyses on three real data sets and one synthetic data set showed that while ignoring negative correlations produced nearly identical results in data sets with relatively few negative correlations, the use of absolute correlations yielded better performance in the data set, which contained a higher proportion of negative correlations. These findings suggest that both positive and negative correlations may carry biologically relevant information, and their treatment should be carefully considered in microbial network clustering. In future work, we aim to explore more comprehensive strategies that can explicitly account for negatively weighted edges and better capture inhibitory or antagonistic interactions within microbial communities.

When evaluating the properties of subnetworks and their nodes, we use various topological property measurement indicators, such as network centrality. Nodes with the following properties can be discovered: a higher degree of connectivity to neighboring nodes, connections to a greater number of paths, closer proximity to other nodes in the network, or higher centrality among adjacent nodes. Therefore, with these indicators, we can identify the central taxa (or hubs) (46, 80, 112) that may play important roles in maintaining the stability of the ecosystem. This step may garner significant interest among researchers because few methods focus on this topic. To assess the topological properties of subnetworks and nodes of several different magnitudes, we used an integrated rank-sum ratio–entropy weight evaluation model (38). This model mitigates the impacts of different indicators and different magnitudes on the comprehensive evaluation of multiple topological properties (113). Furthermore, this model is an objective evaluation method that follows the principle that the greater the disparity in an indicator is, the lower its information entropy and the greater the amount of information it contains (77). If all evaluation objects’ values for a particular indicator are equal, that indicator does not play a role in the comprehensive evaluation (114). This method comprehensively scores and ranks the evaluation objects, facilitating the selection of dominant microbial communities at each site.

The focus of this study was to demonstrate an analytical workflow for microbial network clustering and central subcommunities identification, so the Spearman correlation coefficient, which is widely recognized as a classic measure for assessing the correlations between microbes, was employed to construct the microbial correlation network. Additionally, several other measurement methods, such as SparCC (61), CCLasso (115), and SPIEC-EASI (116), can also be selected and utilized for network construction. Scientists can choose the appropriate method for measuring the correlations of microbes based on their research purpose. Our “MNetClass” R package will continue to be improved and updated in the future, such as by incorporating more association measurement options.

## Data Availability

The raw sequence data reported in this paper have been deposited in the Genome Sequence Archive in the National Genomics Data Center, China National Center for Bioinformation/Beijing Institute of Genomics, Chinese Academy of Sciences (GSA: CRA013444), which are publicly accessible at https://bigd.big.ac.cn/gsa/browse/CRA013444. The open-source MNetClass R package and tutorial are available at GitHub: https://github.com/YihuaWWW/MNetClass.

## SUPPLEMENTAL MATERIAL

Fig. S1 msystems.00989-25-s0001.tif Key subnetworks and central microbes at the tongue.

Fig. S2 msystems.00989-25-s0002.tif Key subnetworks and central microbes at the buccal mucosa.

Fig. S3 msystems.00989-25-s0003.tif Key subnetworks and central microbes at the gingival crevicular fluid.

Fig. S4 msystems.00989-25-s0004.tif Key subnetworks and central microbes at the dental plaque.

Supplemental Material msystems.00989-25-s0005.docx Additional experimental details.

Legends msystems.00989-25-s0006.docx Supplemental figure legends.

Supplemental Tables msystems.00989-25-s0007.xlsx Tables S1 to S7.

ASM does not own the copyrights to Supplemental Material that may be linked to, or accessed through, an article. The authors have granted ASM a non-exclusive, world-wide license to publish the Supplemental Material files. Please contact the corresponding author directly for reuse.

## References

[1] Schupack DA, Mars RAT, Voelker DH, Abeykoon JP, Kashyap PC. 2022. The promise of the gut microbiome as part of individualized treatment strategies. Nat Rev Gastroenterol Hepatol 19:7–25. doi:10.1038/s41575-021-00499-1 34453142 PMC8712374

[2] Fan Y, Pedersen O. 2021. Gut microbiota in human metabolic health and disease. Nat Rev Microbiol 19:55–71. doi:10.1038/s41579-020-0433-9 32887946

[3] Proctor DM, Fukuyama JA, Loomer PM, Armitage GC, Lee SA, Davis NM, Ryder MI, Holmes SP, Relman DA. 2018. A spatial gradient of bacterial diversity in the human oral cavity shaped by salivary flow. Nat Commun 9:681. doi:10.1038/s41467-018-02900-1 29445174 PMC5813034

[4] Hajishengallis G, Lamont RJ, Koo H. 2023. Oral polymicrobial communities: assembly, function, and impact on diseases. Cell Host Microbe 31:528–538. doi:10.1016/j.chom.2023.02.009 36933557 PMC10101935

[5] Wang N, Fang J-Y. 2023. Fusobacterium nucleatum, a key pathogenic factor and microbial biomarker for colorectal cancer. Trends Microbiol 31:159–172. doi:10.1016/j.tim.2022.08.010 36058786

[6] Li R, Liu Y, Zhou F, Yang H, Li J, Dai N, Sun W, Kong J, Gao S. 2023. Clinical significance of Porphyromonas gingivalis enriching cancer stem cells by inhibiting programmed cell death factor 4 in esophageal squamous cell carcinoma. ACS Infect Dis 9:1846–1857. doi:10.1021/acsinfecdis.3c00182 37723647

[7] Guo L, He X, Shi W. 2014. Intercellular communications in multispecies oral microbial communities. Front Microbiol 5:328. doi:10.3389/fmicb.2014.00328 25071741 PMC4076886

[8] Magne F, Gotteland M, Gauthier L, Zazueta A, Pesoa S, Navarrete P, Balamurugan R. 2020. The firmicutes/bacteroidetes ratio: a relevant marker of gut dysbiosis in obese patients? Nutrients 12:1474. doi:10.3390/nu12051474 32438689 PMC7285218

[9] Kolenbrander PE, Andersen RN, Moore LV. 1989. Coaggregation of Fusobacterium nucleatum, Selenomonas flueggei, Selenomonas infelix, Selenomonas noxia, and Selenomonas sputigena with strains from 11 genera of oral bacteria. Infect Immun 57:3194–3203. doi:10.1128/iai.57.10.3194-3203.1989 2777378 PMC260789

[10] Kolenbrander PE, Parrish KD, Andersen RN, Greenberg EP. 1995. Intergeneric coaggregation of oral Treponema spp. with Fusobacterium spp. and intrageneric coaggregation among Fusobacterium spp. Infect Immun 63:4584–4588. doi:10.1128/iai.63.12.4584-4588.1995 7591109 PMC173658

[11] Kolenbrander PE, Andersen RN, Blehert DS, Egland PG, Foster JS, Palmer RJ Jr. 2002. Communication among oral bacteria. Microbiol Mol Biol Rev 66:486–505, doi:10.1128/MMBR.66.3.486-505.2002 12209001 PMC120797

[12] Kaplan CW, Lux R, Haake SK, Shi W. 2009. The Fusobacterium nucleatum outer membrane protein RadD is an arginine-inhibitable adhesin required for inter-species adherence and the structured architecture of multispecies biofilm. Mol Microbiol 71:35–47. doi:10.1111/j.1365-2958.2008.06503.x 19007407 PMC2741168

[13] Lima BP, Shi W, Lux R. 2017. Identification and characterization of a novel Fusobacterium nucleatum adhesin involved in physical interaction and biofilm formation with Streptococcus gordonii. Microbiologyopen 6:e00444. doi:10.1002/mbo3.444 28173636 PMC5458471

[14] Liu T, Yang R, Zhou J, Lu X, Yuan Z, Wei X, Guo L. 2021. Interactions between Streptococcus gordonii and Fusobacterium nucleatum altered bacterial transcriptional profiling and attenuated the immune responses of macrophages. Front Cell Infect Microbiol 11:783323. doi:10.3389/fcimb.2021.783323 35071038 PMC8776643

[15] Strimbu K, Tavel JA. 2010. What are biomarkers? Curr Opin HIV AIDS 5:463–466. doi:10.1097/COH.0b013e32833ed177 20978388 PMC3078627

[16] Wang J, Jia H. 2016. Metagenome-wide association studies: fine-mining the microbiome. Nat Rev Microbiol 14:508–522. doi:10.1038/nrmicro.2016.83 27396567

[17] Rao C, Coyte KZ, Bainter W, Geha RS, Martin CR, Rakoff-Nahoum S. 2021. Multi-kingdom ecological drivers of microbiota assembly in preterm infants. Nature 591:633–638. doi:10.1038/s41586-021-03241-8 33627867 PMC7990694

[18] Belenguer A, Duncan SH, Calder AG, Holtrop G, Louis P, Lobley GE, Flint HJ. 2006. Two routes of metabolic cross-feeding between Bifidobacterium adolescentis and butyrate-producing anaerobes from the human gut. Appl Environ Microbiol 72:3593–3599. doi:10.1128/AEM.72.5.3593-3599.2006 16672507 PMC1472403

[19] Yilmaz B, Juillerat P, Øyås O, Ramon C, Bravo FD, Franc Y, Fournier N, Michetti P, Mueller C, Geuking M, Pittet VEH, Maillard MH, Rogler G, Wiest R, Stelling J, Macpherson AJ, Swiss IBD Cohort Investigators. 2019. Microbial network disturbances in relapsing refractory Crohn’s disease. Nat Med 25:323–336. doi:10.1038/s41591-018-0308-z 30664783

[20] Mac Aogáin M, Narayana JK, Tiew PY, Ali NABM, Yong VFL, Jaggi TK, Lim AYH, Keir HR, Dicker AJ, Thng KX, et al.. 2021. Integrative microbiomics in bronchiectasis exacerbations. Nat Med 27:688–699. doi:10.1038/s41591-021-01289-7 33820995

[21] Kuntal BK, Chandrakar P, Sadhu S, Mande SS. 2019. “NetShift”: a methodology for understanding “driver microbes” from healthy and disease microbiome datasets. ISME J 13:442–454. doi:10.1038/s41396-018-0291-x 30287886 PMC6331612

[22] Xiao L, Zhang F, Zhao F. 2022. Large-scale microbiome data integration enables robust biomarker identification. Nat Comput Sci 2:307–316. doi:10.1038/s43588-022-00247-8 38177817 PMC10766547

[23] McGregor K, Labbe A, Greenwood CMT. 2020. MDiNE: a model to estimate differential co-occurrence networks in microbiome studies. Bioinformatics 36:1840–1847. doi:10.1093/bioinformatics/btz824 31697315 PMC7075537

[24] Peschel S, Müller CL, von Mutius E, Boulesteix A-L, Depner M. 2021. NetCoMi: network construction and comparison for microbiome data in R. Brief Bioinform 22:bbaa290. doi:10.1093/bib/bbaa290 33264391 PMC8293835

[25] Matchado MS, Lauber M, Reitmeier S, Kacprowski T, Baumbach J, Haller D, List M. 2021. Network analysis methods for studying microbial communities: a mini review. Comput Struct Biotechnol J 19:2687–2698. doi:10.1016/j.csbj.2021.05.001 34093985 PMC8131268

[26] Faust K. 2021. Open challenges for microbial network construction and analysis. ISME J 15:3111–3118. doi:10.1038/s41396-021-01027-4 34108668 PMC8528840

[27] Blondel VD, Guillaume J-L, Lambiotte R, Lefebvre E. 2008. Fast unfolding of communities in large networks. J Stat Mech 2008:10008. doi:10.1088/1742-5468/2008/10/P10008

[28] Zhang B, Horvath S. 2005. A general framework for weighted gene co-expression network analysis. Stat Appl Genet Mol Biol 4:Article17. doi:10.2202/1544-6115.1128 16646834

[29] Röttjers L, Faust K. 2020. Manta: a clustering algorithm for weighted ecological networks. mSystems 5:10. doi:10.1128/mSystems.00903-19 PMC7029223 32071163

[30] Feng K, Peng X, Zhang Z, Gu S, He Q, Shen W, Wang Z, Wang D, Hu Q, Li Y, Wang S, Deng Y. 2022. iNAP: an integrated network analysis pipeline for microbiome studies. Imeta 1:e13. doi:10.1002/imt2.13 38868563 PMC10989900

[31] Wen T, Liu Y-X, Liu L, Niu G, Ding Z, Teng X, Ma J, Liu Y, Yang S, Xie P, Zhang T, Wang L, Lu Z, Shen Q, Yuan J. 2025. ggClusterNet 2: an R package for microbial co-occurrence networks and associated indicator correlation patterns. Imeta 4:e70041. doi:10.1002/imt2.70041 40469522 PMC12130565

[32] Röttjers L, Faust K. 2018. From hairballs to hypotheses-biological insights from microbial networks. FEMS Microbiol Rev 42:761–780. doi:10.1093/femsre/fuy030 30085090 PMC6199531

[33] Aldous DJ. 1989. Lower bounds for covering times for reversible Markov chains and random walks on graphs. J Theor Probab 2:91–100. doi:10.1007/BF01048272

[34] Lovász L. 1993. Random walks on graphs. Vol. 2. Combinatorics, Paul erdos is eighty.

[35] Pons P, Latapy M. 2005. Computer and information sciences - ISCIS 2005. 20th International Symposium, Istanbul, Turkey, October 26-28, 2005. Vol. 20, p 284–293, Springer. doi:10.1007/11569596_31

[36] Rosvall M, Bergstrom CT. 2008. Maps of random walks on complex networks reveal community structure. Proc Natl Acad Sci USA 105:1118–1123. doi:10.1073/pnas.0706851105 18216267 PMC2234100

[37] Lieberson S. 1976. Rank-sum comparisons between groups. Sociol Methodol 7:276. doi:10.2307/270713

[38] Lu H, Zhu C, Cao X, Hsu Y. 2022. The sustainability evaluation of masks based on the integrated rank sum ratio and entropy weight method. Sustainability 14:5706. doi:10.3390/su14095706

[39] Liu F, Xue S, Wu J, Zhou C, Hu W, Paris C, Nepal S, Yang J, Yu PS. 2005. Deep learning for community detection: progress, challenges and opportunities. arXiv. doi:10.48550/arXiv.2005.08225

[40] Koschützki D. 2005. Centrality indices, p 16–61. In Network analysis: methodological foundations

[41] Freeman LC. 1977. A set of measures of centrality based on betweenness. Sociometry 40:35. doi:10.2307/3033543

[42] Poudel R, Jumpponen A, Schlatter DC, Paulitz TC, Gardener BBM, Kinkel LL, Garrett KA. 2016. Microbiome networks: a systems framework for identifying candidate microbial assemblages for disease management. Phytopathology 106:1083–1096. doi:10.1094/PHYTO-02-16-0058-FI 27482625

[43] Freeman LC. 2002. Centrality in social networks: conceptual clarification, p 238–263. In Social network: critical concepts in sociology. Vol. 1. Londres: Routledge.

[44] Bonacich P. 1987. Power and centrality: a family of measures. Am J Soc 92:1170–1182. doi:10.1086/228631

[45] Ruhnau B. 2000. Eigenvector-centrality — a node-centrality? Soc Networks 22:357–365. doi:10.1016/S0378-8733(00)00031-9

[46] Junker BH, Schreiber F. 2011. Analysis of biological networks. John Wiley & Sons.

[47] Clauset A, Newman MEJ, Moore C. 2004. Finding community structure in very large networks. Phys Rev E 70:066111. doi:10.1103/PhysRevE.70.066111 15697438

[48] Newman MEJ, Girvan M. 2004. Finding and evaluating community structure in networks. Phys Rev E 69:026113. doi:10.1103/PhysRevE.69.026113 14995526

[49] Guimerà R, Nunes Amaral LA. 2005. Functional cartography of complex metabolic networks. Nature 433:895–900. doi:10.1038/nature03288 15729348 PMC2175124

[50] White DR, Harary F. 2001. The cohesiveness of blocks in social networks: node connectivity and conditional density. Sociol Methodol 31:305–359. doi:10.1111/0081-1750.00098

[51] Watts DJ, Strogatz SH. 1998. Collective dynamics of “small-world” networks. Nature 393:440–442. doi:10.1038/30918 9623998

[52] Badri M, Kurtz ZD, Bonneau R, Müller CL. 2020. Shrinkage improves estimation of microbial associations under different normalization methods. NAR Genom Bioinform 2:lqaa100. doi:10.1093/nargab/lqaa100 33575644 PMC7745771

[53] McMurdie PJ, Holmes S. 2014. Waste not, want not: why rarefying microbiome data is inadmissible. PLoS Comput Biol 10:e1003531. doi:10.1371/journal.pcbi.1003531 24699258 PMC3974642

[54] Daniels HE. 1944. The relation between measures of correlation in the universe of sample permutations. Biometrika 33:129. doi:10.2307/2334112

[55] Spearman C. 1987. The proof and measurement of association between two things. By C. Spearman, 1904. Am J Psychol 100:441–471. 3322052

[56] Layeghifard M, Hwang DM, Guttman DS. 2017. Disentangling interactions in the microbiome: a network perspective. Trends Microbiol 25:217–228. doi:10.1016/j.tim.2016.11.008 27916383 PMC7172547

[57] Guo B, Zhang L, Sun H, Gao M, Yu N, Zhang Q, Mou A, Liu Y. 2022. Microbial co-occurrence network topological properties link with reactor parameters and reveal importance of low-abundance genera. NPJ Biofilms Microbiomes 8:3. doi:10.1038/s41522-021-00263-y 35039527 PMC8764041

[58] Gao C, Xu L, Montoya L, Madera M, Hollingsworth J, Chen L, Purdom E, Singan V, Vogel J, Hutmacher RB, Dahlberg JA, Coleman-Derr D, Lemaux PG, Taylor JW. 2022. Co-occurrence networks reveal more complexity than community composition in resistance and resilience of microbial communities. Nat Commun 13:3867. doi:10.1038/s41467-022-31343-y 35790741 PMC9256619

[59] Barberán A, Bates ST, Casamayor EO, Fierer N. 2012. Using network analysis to explore co-occurrence patterns in soil microbial communities. ISME J 6:343–351. doi:10.1038/ismej.2011.119 21900968 PMC3260507

[60] Faust K, Sathirapongsasuti JF, Izard J, Segata N, Gevers D, Raes J, Huttenhower C. 2012. Microbial co-occurrence relationships in the human microbiome. PLoS Comput Biol 8:e1002606. doi:10.1371/journal.pcbi.1002606 22807668 PMC3395616

[61] Friedman J, Alm EJ. 2012. Inferring correlation networks from genomic survey data. PLoS Comput Biol 8:e1002687. doi:10.1371/journal.pcbi.1002687 23028285 PMC3447976

[62] Jeong H, Tombor B, Albert R, Oltvai ZN, Barabási A-L. 2000. The large-scale organization of metabolic networks. Nature 407:651–654. doi:10.1038/35036627 11034217

[63] Albert R. 2005. Scale-free networks in cell biology. J Cell Sci 118:4947–4957. doi:10.1242/jcs.02714 16254242

[64] Tong M, Li X, Wegener Parfrey L, Roth B, Ippoliti A, Wei B, Borneman J, McGovern DPB, Frank DN, Li E, Horvath S, Knight R, Braun J. 2013. A modular organization of the human intestinal mucosal microbiota and its association with inflammatory bowel disease. PLoS One 8:e80702. doi:10.1371/journal.pone.0080702 24260458 PMC3834335

[65] Langfelder P, Horvath S. 2008. WGCNA: an R package for weighted correlation network analysis. BMC Bioinformatics 9:1–13. doi:10.1186/1471-2105-9-559 19114008 PMC2631488

[66] Ning W, Acharya A, Li S, Schmalz G, Huang S. 2022. Identification of key pyroptosis-related genes and distinct pyroptosis-related clusters in periodontitis. Front Immunol 13:862049. doi:10.3389/fimmu.2022.862049 35844512 PMC9281553

[67] Clauset A, Shalizi CR, Newman MEJ. 2009. Power-law distributions in empirical data. SIAM Rev 51:661–703. doi:10.1137/070710111

[68] Girvan M, Newman MEJ. 2002. Community structure in social and biological networks. Proc Natl Acad Sci USA 99:7821–7826. doi:10.1073/pnas.122653799 12060727 PMC122977

[69] Faust K, Raes J. 2012. Microbial interactions: from networks to models. Nat Rev Microbiol 10:538–550. doi:10.1038/nrmicro2832 22796884

[70] Bhatia V, Rani R. 2018. DFuzzy: a deep learning-based fuzzy clustering model for large graphs. Knowl Inf Syst 57:159–181. doi:10.1007/s10115-018-1156-3

[71] Aldous D, Fill J. 2002. Reversible Markov chains and random walks on graphs.

[72] Meng Q, Liu S, Guo Y, Hu Y, Yu Z, Bello A, Wang Z, Xu W, Xu X. 2023. The co-occurrence network patterns and keystone species of microbial communities in cattle manure-corn straw composting. Environ Sci Pollut Res Int 30:20265–20276. doi:10.1007/s11356-022-23599-0 36251182

[73] Dasgupta A, Kumar R, Sarlos T. 2014. On estimating the average degree. In proceedings of the 23rd international conference on World Wide Web (WWW'14). , p 795–806 Association for Computing Machinery, New York, NY, USA

[74] Banerjee S, Schlaeppi K, van der Heijden MGA. 2018. Keystone taxa as drivers of microbiome structure and functioning. Nat Rev Microbiol 16:567–576. doi:10.1038/s41579-018-0024-1 29789680

[75] Bohannan BJ, Relman DA. The application of ecological theory toward an understanding of the human microbiome. 10.1126/science.1224203 PMC4208626 22674335

[76] Li F, Chen L, Zhang J, Yin J, Huang S. 2017. Bacterial community structure after long-term organic and inorganic fertilization reveals important associations between soil nutrients and specific taxa involved in nutrient transformations. Front Microbiol 8:187. doi:10.3389/fmicb.2017.00187 28232824 PMC5298992

[77] Zhu Y, Tian D, Yan F. 2020. Effectiveness of entropy weight method in decision-making. Math Probl Eng 2020:1–5. doi:10.1155/2020/3564835

[78] Johnson RA, Miller I, Freund JE. 2000. Probability and statistics for engineers.

[79] Strogatz SH. 2001. Exploring complex networks. Nature 410:268–276. doi:10.1038/35065725 11258382

[80] Agler MT, Ruhe J, Kroll S, Morhenn C, Kim S-T, Weigel D, Kemen EM. 2016. Microbial hub taxa link host and abiotic factors to plant microbiome variation. PLoS Biol 14:e1002352. doi:10.1371/journal.pbio.1002352 26788878 PMC4720289

[81] Wang Q, Cole JR. 2024. Updated RDP taxonomy and RDP Classifier for more accurate taxonomic classification. Microbiol Resour Announc 13:e0106323. doi:10.1128/mra.01063-23 38436268 PMC11008197

[82] Hochreiter S, Bodenhofer U, Heusel M, Mayr A, Mitterecker A, Kasim A, Khamiakova T, Van Sanden S, Lin D, Talloen W, Bijnens L, Göhlmann HWH, Shkedy Z, Clevert D-A. 2010. FABIA: factor analysis for bicluster acquisition. Bioinformatics 26:1520–1527. doi:10.1093/bioinformatics/btq227 20418340 PMC2881408

[83] Xu Y, Wang Y, Xu J, Song Y, Liu B, Xiong Z. 2022. Leveraging existing 16SrRNA microbial data to define a composite biomarker for autism spectrum disorder. Microbiol Spectr 10. doi:10.1128/spectrum.00331-22 PMC9431227 35762814

[84] Liu S, Wang Y, Zhao L, Sun X, Feng Q. 2020. Microbiome succession with increasing age in three oral sites. Aging (Milano) 12:7874–7907. doi:10.18632/aging.103108 PMC7244077 32379704

[85] Liu Z, Sun Y, Ma A, Wang X, Xu D, Spakowics D, Ma Q, Liu B. 2024. An explainable graph neural framework to identify cancer-associated intratumoral microbial communities. Adv Sci. doi:10.1101/2023.04.16.537088 PMC11538693 39225619

[86] Grandini M, Bagli E, Visani G. 2020. Metrics for multi-class classification: an overview. arXiv. doi:10.48550/arXiv.2008.05756

[87] Yabuuchi S, Oiki S, Minami S, Takase R, Watanabe D, Hashimoto W. 2022. Enhanced propagation of Granulicatella adiacens from human oral microbiota by hyaluronan. Sci Rep 12:10948. doi:10.1038/s41598-022-14857-9 35768476 PMC9243090

[88] Karched M, Bhardwaj RG, Asikainen SE. 2015. Coaggregation and biofilm growth of Granulicatella spp. with Fusobacterium nucleatum and Aggregatibacter actinomycetemcomitans. BMC Microbiol 15:114. doi:10.1186/s12866-015-0439-z 26025449 PMC4448563

[89] Leo JC. 2025. Interaction between bacterial adhesins leads to coaggregation by the oral bacteria Veillonella parvula and Streptococcus gordonii. MBio 16:e0327924. doi:10.1128/mbio.03279-24 39791898 PMC11796380

[90] Palmer RJ, Shah N, Valm A, Paster B, Dewhirst F, Inui T, Cisar JO. 2017. Interbacterial adhesion networks within early oral biofilms of single human hosts. Appl Environ Microbiol 83:e00407-17. doi:10.1128/AEM.00407-17 28341674 PMC5440702

[91] Hughes CV, Kolenbrander PE, Andersen RN, Moore LV. 1988. Coaggregation properties of human oral Veillonella spp.: relationship to colonization site and oral ecology. Appl Environ Microbiol 54:1957–1963. doi:10.1128/aem.54.8.1957-1963.1988 3178207 PMC202786

[92] Brohée S, van Helden J. 2006. Evaluation of clustering algorithms for protein-protein interaction networks. BMC Bioinformatics 7:1–19. doi:10.1186/1471-2105-7-488 17087821 PMC1637120

[93] Banerjee S, Kirkby CA, Schmutter D, Bissett A, Kirkegaard JA, Richardson AE. 2016. Network analysis reveals functional redundancy and keystone taxa amongst bacterial and fungal communities during organic matter decomposition in an arable soil. Soil Biol Biochem 97:188–198. doi:10.1016/j.soilbio.2016.03.017

[94] Jiang Y, Li S, Li R, Zhang J, Liu Y, Lv L, Zhu H, Wu W, Li W. 2017. Plant cultivars imprint the rhizosphere bacterial community composition and association networks. Soil Biol Biochem 109:145–155. doi:10.1016/j.soilbio.2017.02.010

[95] Berry D, Widder S. 2014. Deciphering microbial interactions and detecting keystone species with co-occurrence networks. Front Microbiol 5:219. doi:10.3389/fmicb.2014.00219 24904535 PMC4033041

[96] Morita E, Narikiyo M, Nishimura E, Yano A, Tanabe C, Sasaki H, Hanada N. 2004. Molecular analysis of age-related changes of Streptococcus anginosus group and Streptococcus mitis in saliva. Oral Microbiol Immunol 19:386–389. doi:10.1111/j.1399-302x.2004.00173.x 15491464

[97] Caufield PW, Dasanayake AP, Li Y, Pan Y, Hsu J, Hardin JM. 2000. Natural history of Streptococcus sanguinis in the oral cavity of infants: evidence for a discrete window of infectivity. Infect Immun 68:4018–4023. doi:10.1128/IAI.68.7.4018-4023.2000 10858217 PMC101685

[98] Ge Y, Caufield PW, Fisch GS, Li Y. 2008. Streptococcus mutans and Streptococcus sanguinis colonization correlated with caries experience in children. Caries Res 42:444–448. doi:10.1159/000159608 18832831 PMC2680676

[99] Bloch S, Hager-Mair FF, Andrukhov O, Schäffer C. 2024. Oral streptococci: modulators of health and disease. Front Cell Infect Microbiol 14:1357631. doi:10.3389/fcimb.2024.1357631 38456080 PMC10917908

[100] Mashima I, Nakazawa F. 2015. Interaction between Streptococcus spp. and Veillonella tobetsuensis in the early stages of oral biofilm formation. J Bacteriol:197. doi:10.1128/jb.02512-14 PMC4455269 25917902

[101] Stinson MW, Safulko K, Levine MJ. 1991. Adherence of Porphyromonas (Bacteroides) gingivalis to Streptococcus sanguis in vitro. Infect Immun 59:102–108. doi:10.1128/iai.59.1.102-108.1991 1987021 PMC257711

[102] Mashima I, Nakazawa F. 2014. The influence of oral Veillonella species on biofilms formed by Streptococcus species. Anaerobe 28:54–61. doi:10.1016/j.anaerobe.2014.05.003 24862495

[103] Kolenbrander PE, Andersen RN, Holdeman LV. 1985. Coaggregation of oral Bacteroides species with other bacteria: central role in coaggregation bridges and competitions. Infect Immun 48:741–746. doi:10.1128/iai.48.3.741-746.1985 3888842 PMC261248

[104] Smith DJ, Anderson JM, King WF, van Houte J, Taubman MA. 1993. Oral streptococcal colonization of infants. Oral Microbiol Immunol 8:1–4. doi:10.1111/j.1399-302x.1993.tb00535.x 8510978

[105] Lang C, Böttner M, Holz C, Veen M, Ryser M, Reindl A, Pompejus M, Tanzer JM. 2010. Specific Lactobacillus/mutans Streptococcus co-aggregation. J Dent Res 89:175–179. doi:10.1177/0022034509356246 20042742

[106] Keller MK, Hasslöf P, Stecksén-Blicks C, Twetman S. 2011. Co-aggregation and growth inhibition of probiotic lactobacilli and clinical isolates of mutans streptococci: an in vitro study. Acta Odontol Scand 69:263–268. doi:10.3109/00016357.2011.554863 21306197

[107] Hardie JM, Whiley RA. 2006. The genus Streptococcus--oral. Prokaryotes 4:76–107. doi:10.1007/0-387-30744-3_2

[108] Hao S, Wang J, Wang Y. 2021. Effectiveness and safety of Bifidobacterium in preventing dental caries: a systematic review and meta-analysis. Acta Odontol Scand 79:613–622. doi:10.1080/00016357.2021.1921259 33956564

[109] Schwendicke F, Horb K, Kneist S, Dörfer C, Paris S. 2014. Effects of heat-inactivated Bifidobacterium BB12 on cariogenicity of Streptococcus mutans in vitro. Arch Oral Biol 59:1384–1390. doi:10.1016/j.archoralbio.2014.08.012 25214308

[110] Widyarman AS, Yunita ST, Prasetyadi T. 2018. Consumption of yogurt containing probiotic Bifidobacterium lactis reduces Streptococcus mutans in orthodontic patients. Sci Dent J 2:19. doi:10.26912/sdj.v2i1.1913

[111] Çömlekcioğlu U, Jezierska S, Opsomer G, Pascottini OB. 2024. Uterine microbial ecology and disease in cattle: a review. Theriogenology 213:66–78. doi:10.1016/j.theriogenology.2023.09.016 37804686

[112] Csardi G, Nepusz T. 1695. The igraph software package for complex network research. Int J Complex Systems 1695:1–9.

[113] Wen S, Su B, Wang Y, Zhai J, Sun H, Chen Z, Huang J, Wang A, Jiang T. 2020. Comprehensive evaluation of hydrological models for climate change impact assessment in the Upper Yangtze River Basin, China. Clim Change 163:1207–1226. doi:10.1007/s10584-020-02929-6

[114] Liu L, Zhou J, An X, Zhang Y, Yang L. 2010. Using fuzzy theory and information entropy for water quality assessment in Three Gorges region, China. Expert Syst Appl 37:2517–2521. doi:10.1016/j.eswa.2009.08.004

[115] Fang H, Huang C, Zhao H, Deng M. 2015. CCLasso: correlation inference for compositional data through Lasso. Bioinformatics 31:3172–3180. doi:10.1093/bioinformatics/btv349 26048598 PMC4693003

[116] Kurtz Z, Mueller C, Miraldi E, Bonneau R. 2017. SpiecEasi: sparse inverse covariance for ecological statistical inference. R package version 1

