# Supplementary figures and images for: MNetClass: a control-free microbial network clustering framework for identifying central subcommunities across ecological niches
Source: mSystems. 2025 Nov 13;10(12):e00989-25. doi: 10.1128/msystems.00989-25 (PMC12710344; doi:10.1128/msystems.00989-25)

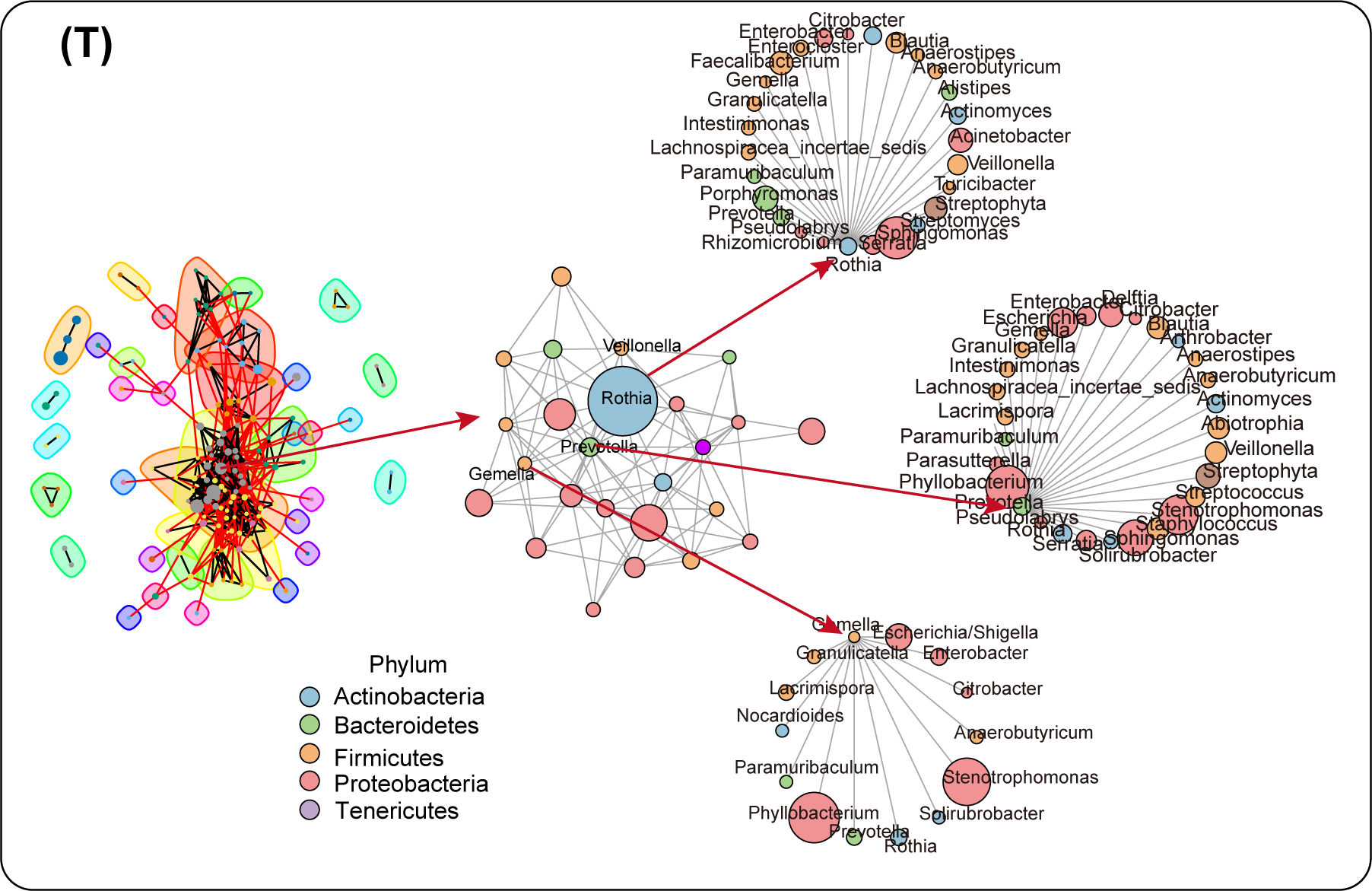

Supplement: Fig. S1 — Key subnetworks and central microbes at the tongue. [file msystems.00989-25-s0001.tif]

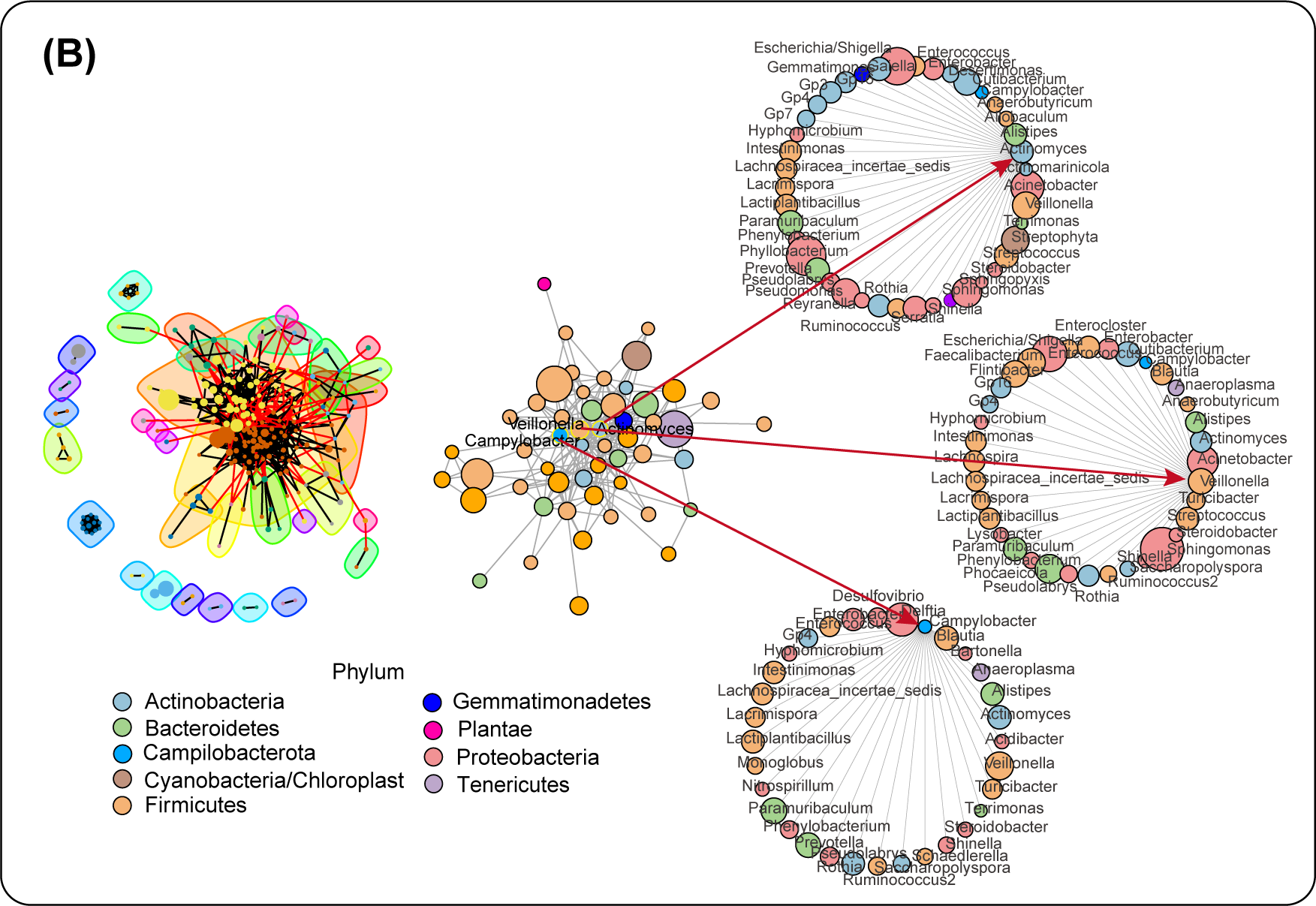

Supplement: Fig. S2 — Key subnetworks and central microbes at the buccal mucosa. [file msystems.00989-25-s0002.tif]

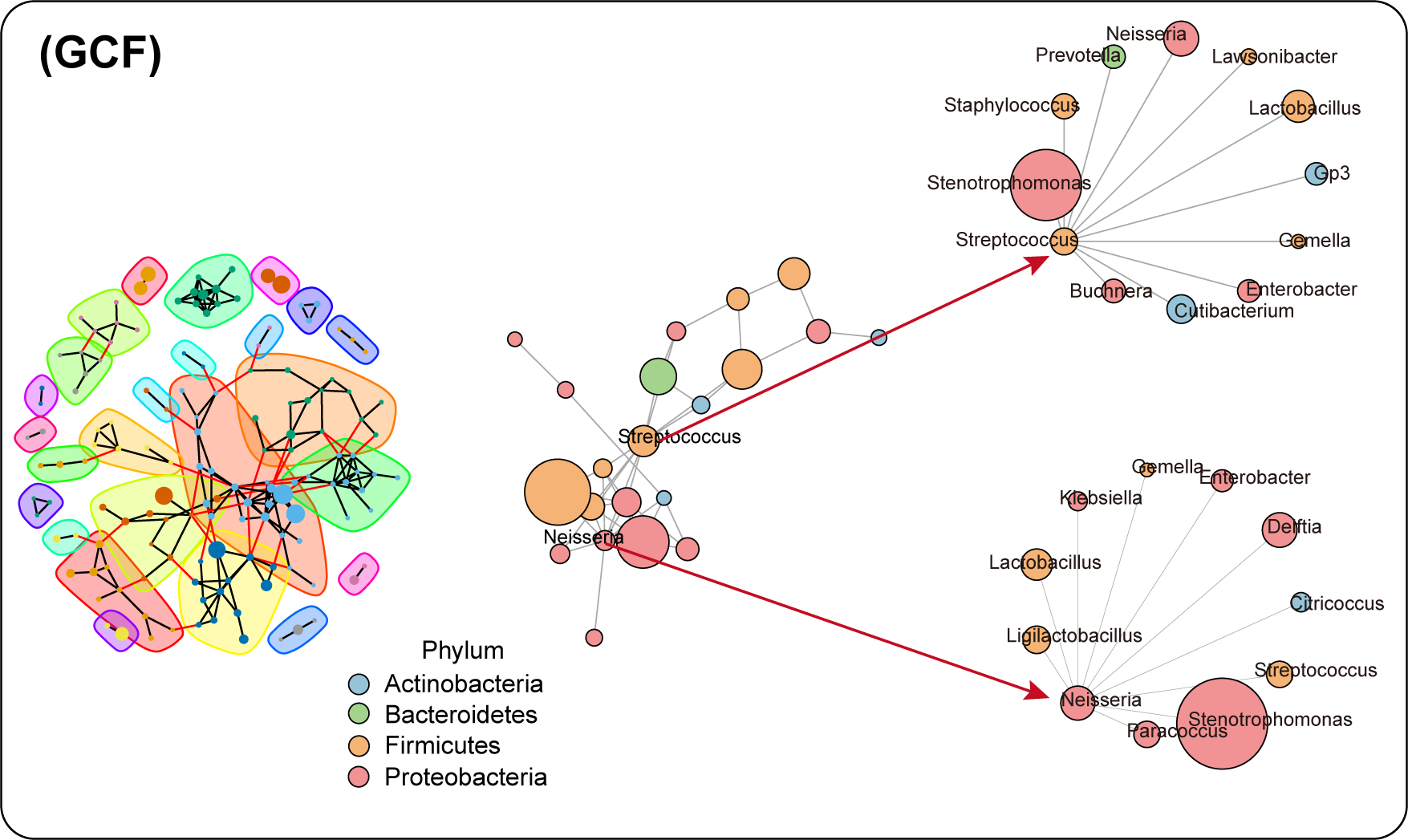

Supplement: Fig. S3 — Key subnetworks and central microbes at the gingival crevicular fluid. [file msystems.00989-25-s0003.tif]

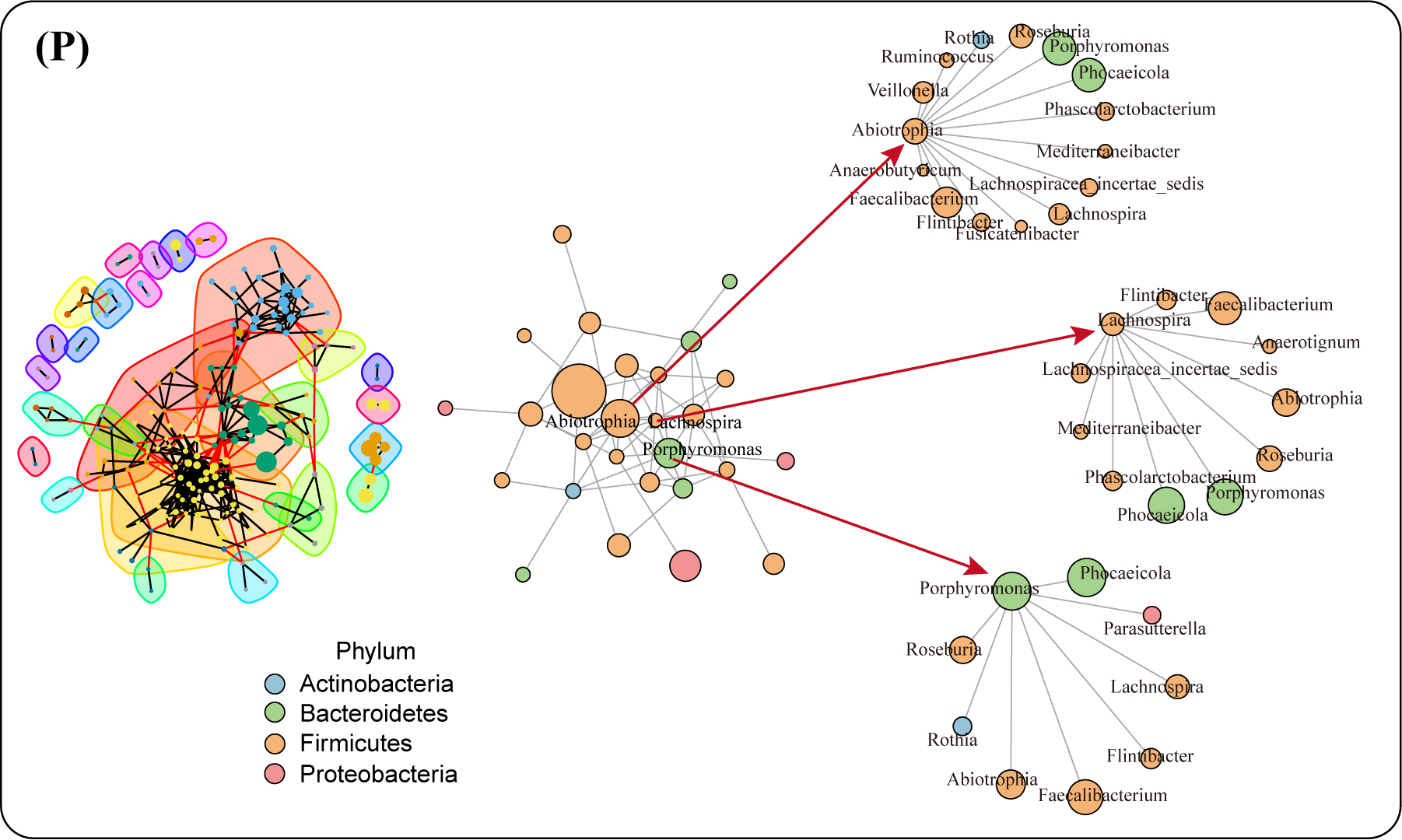

Supplement: Fig. S4 — Key subnetworks and central microbes at the dental plaque. [file msystems.00989-25-s0004.tif]
