# Supplementary material for: MNetClass: a control-free microbial network clustering framework for identifying central subcommunities across ecological niches
Source: mSystems. 2025 Nov 13;10(12):e00989-25. doi: 10.1128/msystems.00989-25 (PMC12710344; doi:10.1128/msystems.00989-25)
Supplement: Supplemental Material — Additional experimental details. [file msystems.00989-25-s0005.docx]

1. **Community detection methods**

| **Community detection** | **Implementation** | **Description** |
| --- | --- | --- |
| Walktrap clustering^1^ | Walktrap clustering using the R function walktrap.community() from igraph package. | - Based on similarity - It computes the probabilities of random walks to derive a measure of similarity (or distance) between different communities. |
| Hierarchical clustering | - Hierarchical clustering using the R function hclust() from stats package ^2^. - The cutree() function (stats) is used for cutting the resulting tree. | - Based on dissimilarity - It creates a hierarchy of clusters where each node starts as its own cluster, and pairs of clusters are merged as one moves up the hierarchy. |
| Modularity clustering^3^ | Modularity clustering using the R function clusters() from igraph package. | - Based on similarity - It aims to optimize the modularity value, a scalar value between -1 and 1 that measures the density of links inside communities as compared to links between communities. |
| Fast greedy modularity  optimization^4^ | Fast greedy modularity  optimization using the R function fastgreedy.community() from igraph package. | - Based on similarity - It is a hierarchical agglomeration algorithm: it starts with each vertex in its own community and iteratively merges communities to maximize the gain in modularity. |
| Clustering based on edge  betweenness^5^ | Clustering based on edge  betweenness using the R function edge.betweenness.community() from igraph package. | - Based on dissimilarity - It detects communities by progressively removing edges from the original graph and recalculates the betweenness of all remaining edges after each removal. |

1. **Undirected weighted network global and node topology property indicators.**

Let $G = (V, E, W)$ with $n=|V|$ vertices, $m=|E|$ edges and $W$ as the set of edge weights be a simple, finite, undirected, weighted graph.

| **Measure** | **Description** | **Representation** | **Application** |
| --- | --- | --- | --- |
| Global network metrics | | | |
| Graph diameter | The maximum value of all shortest paths within a network^6^. | 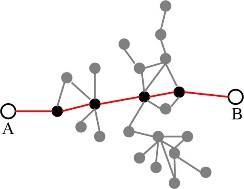  In the schematic diagram, the shortest path is defined as the path between points A and B where the total weight (i.e., the sum of the weights of all edges in the path) is minimized. | Reflecting the scale of the network. |
| Graph density | In a network, the ratio of the actual number of edges to the potential number of edges is presented^7^. | $den(G)=\frac{\left\vert E_{G} \right\vert}{\left\vert V_{G} \right\vert(\left\vert V_{G} \right\vert-1)/2}$; | Highlighting the network's cohesiveness characteristics. |
| Average path length | The arithmetic mean of all shortest paths between nodes in a network^6^. | $\bar{h}=\frac{1}{\vert V\vert(\vert V\vert-1)}\sum_{i\neq j} h_{ij}$ Where, $h_{ij}$ is the length of the shortest path between *i* and *j*, calculated based on the edge weights. | Representing the global efficiency and vulnerability of the network, |
| Average degree | The average degree of all nodes in a network^6^. | $AVD=\frac{\sum_{i=1}^{\vert G\vert} deg(v_{i})}{\vert V\vert}=\frac{2\left\vert E \right\vert}{\left\vert V \right\vert}$ Where, $deg(v_{i})$ is the degree of vertex $i$ in graph *G.* | Signifying the network's density and robustness. |
| Edge connectivity | The minimum number of edges/vertex required to disconnect a network^6^. This metric is not applicable to a fully connected network. | 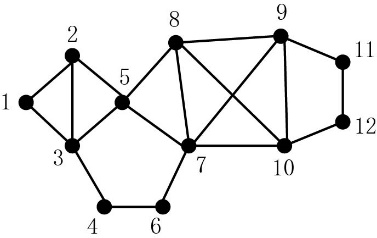  is edge connectivity and $\kappa(G)$ is vertex connectivity of a graph $G$. In the example graph, $\kappa'(G)=2$ (e.g., removal of edges (9,11) and (10,12) would disconnect the graph) and $\kappa(G)=2$ (e.g., removal of vertices 3 and 5 would disconnected the graph). | Illuminating the connectivity characteristics of the network. |
| Vertex connectivity |
| Clustering coefficient | The clustering coefficient of an undirected graph is a measure of the number of triangles within the graph^8^, considering edge weights. | $C_{global}=\frac{\sum_{triangles} w_{triangle}}{\sum_{triplets} w_{triplet}}$ Where, $w_{triangle}$is the weight of a triangle; $w_{triplet}$is the weight of a triplet. | Representing an assessment of the network's global clustering tendency. |
| Modularity | Modularity indicates the division of the network into communities, characterized by numerous edges within communities and fewer between them^4^. | Let $C$ denote a partition of the graph's nodes into communities, where each node $i$ belongs to a community $c_{i}$​. The weighted modularity of the partition $C$ is defined as: $Q_{w}=\frac{1}{2m}\sum_{i,j} (A_{ij}-\frac{k_{i}k_{j}}{2m})\delta(c_{i}, c_{j})$ Where $A_{ij}$​ is the weight of the edge between nodes $i$ and $j$; $k_{i}$​ and $k_{j}$​ are the weighted degrees of nodes $i$ and $j$, respectively; $m=\sum_{i,j} A_{i,j}$ is the total weight of all edges in the graph; $\delta(c_{i}, c_{j})$ is the Kronecker delta function, which equals 1 if nodes $i$ and $j$ are in the same community and 0 otherwise. | Evaluating the quality of the network's community partitioning |
| Nodes metrics | | | |
| Degree centrality | The number of adjacent nodes^9^. | $\mathcal{C}_{deg}\left( v \right)=\sum_{\mu\in N(v)} \omega(v,\mu)$ Where, $N(v)$ is the set of neighbors (adjacent nodes) of node $v$; $\omega(v,\mu)$ is the weight of the edge between node $v$ and its neighbor $\mu$. | Nodes with more connections tend to exert greater influence in a network. For instance, in a social network, individuals with more friends wield more influence, just as species with more associated microbes in a microbial association network are deemed more influential based on this property. |
| Betweenness centrality | The probability that a node lies on the shortest path between all other nodes^9^. Central nodes possess the capacity to connect subnetworks^10^. | $c_{B}(v)=\sum_{s\neq t\neq v\in V} \frac{\sigma(s,t\vert v)}{\sigma(s,t)}$ where, $\sigma(s,t\vert v)$ is the number of shortest paths through vertex $v$ between $s$ and $t$, $\text{σ(}\text{s,t}\text{)}$ is the total number of shortest paths between $s$ and $t$ (whether passing $v$ or not). The shortest path is calculated based on the edge weights.  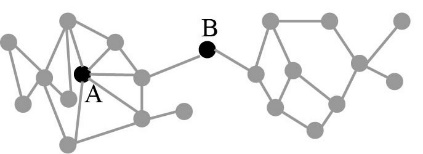  In the example figure, while vertex $v_{A}$ has a high degree, its removal does not necessarily affect communication within the network. However, removal of vertices with low degree may have significant effects on communication or mass flow within the network, as seen for vertex $v_{B}$. | This represents the significance of a node's position within network paths. If these paths are considered channels for communication, then nodes situated on multiple paths become critical junctures in the communication process. |
| Closeness centrality | The inverse sum of the shortest paths between a node and all other nodes defines its closeness centrality^9^. Nodes with the highest closeness centrality exhibit the shortest paths to all other nodes. | Let $G=(V,E)$be an undirected and connected network. The closeness^11^ is defined as $\mathcal{C}_{clo}(s) :=\frac{1}{\sum_{t\in V} dist(s,t)}$ Where $dist(s, t)$ is the length of a shortest path (in terms of edge weights) between $s$ and $t$*.*  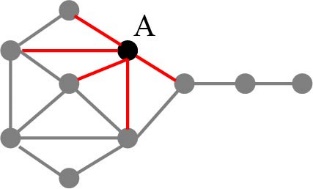  In the example figure, vertex $v_{B}$ has the highest closeness centrality value. | Reflecting the proximity of a particular node to others in the network. In essence, the closer a node is to other nodes, the greater its closeness centrality, denoting its significance or "importance". In a microbial community context, when identifying key microbes, it's desirable for the key microbe to have direct or indirect associations with as many other microbes as possible. In the constructed microbial association network, nodes representing key microbes should ideally be in close proximity to other nodes, thereby exhibiting high closeness centrality. |
| Eigenvector centrality | Eigenvector centrality is a measure of the importance of a node within the network based on the weights of its connections and the importance of its neighbors^12,13^. If a node, and its associated nodes, occupies a central position within the network, then it is considered a central node. | The eigenvector centrality^14^ is the eigenvector $\vec{\mathcal{C}_{\mathrm{eiv}}}$ of the largest eigenvalue $\lambda_{max}$ in absolute value of the following equation system: $\lambda\vec{\mathcal{C}_{\mathrm{eiv}}}=A\vec{\mathcal{C}_{\mathrm{eiv}}}$ where, $A$ the weighted adjacency matrix for $G$*.*  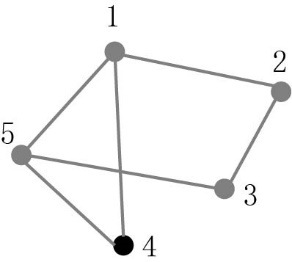  share the same degree, yet $v_{4}$ have a higher eigenvector centrality. This can be attributed to the fact that $v_{4}$ connects the two nodes $v_{1},v_{5}$ with the largest centrality. The example figure reflects that if the neighbor centrality of a node is higher, the centrality of the node itself is also higher | Here, centrality measures often derive from concepts related to "status", "prestige", or "rank". The underlying principle of eigenvector centrality posits: a node's centrality increases with the centrality of its neighbors. Within a microbial community, if microbes associated with a particular microbe also have high associations with others, it suggests that this microbe holds significant importance within the community. |

1. **Dataset**

**Ethical Statement and Sample Collection**

This study was approved by the Ethics Committee of Shandong University School of Stomatology (Approval No.: R20180504). Prior to inclusion in the study, informed consent was obtained from all volunteers. All samples were collected in accordance with the following guidelines and protocols. Saliva specimens were collected 2 hours after meals: prior to saliva collection, participants rinsed their mouths three times with sterile double distilled water, and then saliva samples were collected using a 1.5 μL centrifuge tube. In order to collect gingival crevicular fluid (GCF) samples, supragingival plaque was removed, and two paper points (Henry Schein, Almere, Netherlands) were inserted into the bottom of the gingival pockets for 10 seconds. The paper points were then immersed in sterile phosphate buffer saline. For tongue dorsum samples, liquid from the soft tissue on the tongue dorsum was collected using a cotton swab, and the swab was immersed in sterile phosphate buffer saline. All samples were immediately transported to the laboratory and stored at -80°C for future use within 20 minutes.

**DNA Extraction, Library Construction, and Sequencing**

Extraction of total DNA was performed using the CTAB/SDS method. The DNA concentration and purity were assessed using 1% agarose gel electrophoresis. Based on the obtained DNA concentration, the DNA was diluted to 1 ng/μl using sterile water. Genomic DNA extracted from all samples was used as the PCR template for library construction and sequencing. Primers targeting the V3-V4 region of the 16S rDNA gene were employed, with the forward primer sequence being 5'-ACTCCTACGGGAGGCAGCA-3' and the reverse primer sequence being 5'-GGACTACHVGGGTWTCTAAT-3'. All PCR reactions were conducted in a total volume of 30 μl, including 15 μl of Phusion High-Fidelity PCR Master Mix (New England Biolabs), 0.2 μM of each forward and reverse primer, and approximately 10 ng of template DNA. The thermal cycling conditions comprised an initial denaturation step at 95℃ for 5 minutes, followed by 30 cycles of denaturation at 95℃ for 30 seconds, annealing at 50℃ for 30 seconds, and extension at 72℃ for 40 seconds. The final extension step was carried out at 72℃ for 7 minutes. The PCR products were subsequently purified using the GeneJET Gel Extraction Kit (Thermo Scientific) and validated through 2% agarose gel electrophoresis. Samples displaying a single amplification product were selected for further experiments. DNA libraries were sequenced by Novogene (Beijing, China) on the Illumina HiSeq 2500 platform.

**Data processing**

The raw sequencing sequences were filtered and quality-controlled using the second-generation microbial bioinformatics platform QIIME2 version. DADA2 is based on sequencing error correction algorithms and generates 5806 exact amplicon sequence variants (ASVs). Subsequently, the obtained ASVs were compared against the ribosomal database project (RDP version 2.14^15^) for taxonomy assignment, resulting in the identification of 810 genera, and 37 phyla. (Key details are described in the Methods section.)

1. **Supplementary methods**

**Spearman correlation analysis among all microbes in a given niche**

For a given set of n samples with paired relative abundance data for taxa $X$ and

taxa $Y$, $\left\{ \left( x_{1},y_{1} \right), \left( x_{2}, y_{2} \right),\cdots,\left( x_{n},y_{n} \right) \right\}$, the Spearman correlation coefficient $r_{s}$ is defined as16 $r_{s}=\frac{cov\left( rg_{X}, rg_{Y} \right)}{\sigma_{rg_{X}}\sigma_{rg_{Y}}}$, where $rg_{X}$and $rg_{Y}$ represent the ranks of the relative abundance data for taxa $X$ and $Y$, respectively. $cov(*, *)$ denotes the covariance of the rank variables, and $\sigma_{rg_{x}}$ and $\sigma_{rg_{Y}}$ represent the standard deviations of the rank variables $rg_{X}$ and $rg_{Y}$.

1. **Evaluating microbial topological properties of subnetworks and central node microbes**

For each oral site, the top 20% of subnetworks (k=20) in terms of node count were selected and evaluated by ten kinds of network topological property indicators, which are shown in Supplementary Table 4. Here, the analysis of subnetworks and central microbes at the niche of the tongue and saliva is detailed as example.

**Evaluating and scoring of microbial subnetworks in the Tongue (T) Region**

**(i)** The original evaluation matrix is constructed based on the scores of the ten indicators for the eight subnetworks, as shown in Supplementary Table 4-5. The matrix is presented below:

$$X=\left[ \begin{matrix} 25 & 105 & 2.499 & 0.350 & 8.400 & 2 & 2 & 0.511 & 0.214 & 1.456 \\ 25 & 116 & 2.618 & 0.387 & 9.280 & 2 & 2 & 0.565 & 0.173 & 1.411 \\ 8 & 14 & 2.483 & 0.500 & 3.500 & 1 & 1 & 0.628 & 0.130 & 1.337 \\ 8 & 19 & 1.720 & 0.679 & 4.750 & 3 & 3 & 0.695 & 0.011 & 1.134 \\ 7 & 12 & 2.549 & 0.571 & 3.429 & 1 & 1 & 0.600 & 0.080 & 1.258 \\ 6 & 10 & 1.758 & 0.667 & 3.333 & 2 & 2 & 0.577 & 0.020 & 1.135 \\ 6 & 7 & 2.562 & 0.467 & 2.333 & 1 & 1 & 0.500 & 0.204 & 1.395 \\ 5 & 5 & 2.449 & 0.500 & 2.000 & 1 & 1 & 0.000 & 0.080 & 1.322 \end{matrix} \right]$$

**(ii)** The subnetwork topological property metrics are standardized using Equation (1). The detailed results are presented below:

$$Z=\left[ \begin{matrix} 1.000 & 0.904 & 0.926 & 0.000 & 0.889 & 0.500 & 0.500 & 0.511 & 1.000 & 0.000 \\ 1.000 & 1.000 & 1.000 & 0.056 & 1.000 & 0.500 & 0.500 & 0.565 & 0.906 & 0.099 \\ 0.227 & 0.105 & 0.917 & 0.231 & 0.273 & 0.000 & 0.000 & 0.628 & 0.808 & 0.262 \\ 0.227 & 0.149 & 0.445 & 0.505 & 0.430 & 1.000 & 1.000 & 0.695 & 0.535 & 0.706 \\ 0.182 & 0.088 & 0.958 & 0.341 & 0.264 & 0.000 & 0.000 & 0.600 & 0.693 & 0.436 \\ 0.136 & 0.044 & 0.966 & 0.179 & 0.126 & 0.000 & 0.000 & 0.500 & 0.978 & 0.135 \\ 0.136 & 0.070 & 0.468 & 0.487 & 0.252 & 0.500 & 0.500 & 0.577 & 0.555 & 0.705 \\ 0.091 & 0.026 & 0.896 & 0.231 & 0.084 & 0.000 & 0.000 & 0.000 & 0.693 & 0.294 \end{matrix} \right]$$

**(iii)** The Rank $R_{ij}$​ of each metric of subnetwork topological properties is calculated. Among the ten network topological property indicators, nine positive metrics are arranged in ascending order. The final indicator, “average path length,” is a negative metric and arranged in descending order. Identical values for the same metric are given average ranks, producing the rank matrix $R$. The results are provided below:

$$R=\left[ \begin{matrix} 6 & 9 & 7 & 1 & 8 & 2 & 2 & 3 & 10 & 1 \\ 6 & 10 & 10 & 2 & 9 & 2 & 2 & 4 & 8 & 2 \\ 5 & 7 & 6 & 4 & 6 & 1 & 1 & 7 & 7 & 4 \\ 5 & 8 & 2 & 8 & 7 & 3 & 3 & 8 & 3 & 9 \\ 4 & 6 & 8 & 5 & 5 & 1 & 1 & 6 & 5 & 6 \\ 3 & 4 & 9 & 3 & 3 & 1 & 1 & 2 & 9 & 3 \\ 3 & 5 & 4 & 7 & 4 & 2 & 2 & 5 & 4 & 8 \\ 2 & 3 & 5 & 4 & 2 & 1 & 1 & 1 & 6 & 5 \end{matrix} \right]$$

**(iv)** Weights are determined using EWM. Based on Equation (4), the information entropy $e_{j}$ and entropy weight $W_{j}$ for ten evaluation metrics are calculated. The results are presented in Supplementary Table 5-1.

**(v)** The $WRSR$ values for the eight subnetworks are computed using Equation (5), with the results provided below:

$$WRSR=\left[ 0.520,0.572,0.455,0.558,0.423,0.325,0.400,0.257 \right]$$

**(vi)** Distribution statistics of $WRSR$ are identified. In this step, the frequency, cumulative frequency, average rank, downward cumulative rating, and probability unit are tabulated in Supplementary Table 5-1. A higher rank indicates a better evaluation of the subject.

**(vii)** With the $Probit$ values corresponding to cumulative frequencies as the independent variable and $WRSR$ values as the dependent variable, the regression equation is computed using the least squares method: $WR\hat{S}R=-0.1779+0.0956\times Probit$，$R^{2}=0.987, F=444.1, P=7.44e-7, df=6$.

**(viii)** The topological properties of the subnetworks are ranked based on $Probit$ and $WR\hat{S}R$, with the results presented in Supplementary Table 5-1.

**Evaluating and scoring of central node microbes at Dorsal Tongue site**
  Similar to the evaluation of subnetwork composite topological properties, we employ an integrated rank-sum ratio–entropy weight evaluation model to evaluate the comprehensive topological attributes of all nodes within subnetworks 8. This decision is based on the scores of five indicators for nodes within the excellent subnetworks 8, as outlined in Supplementary Material 2. Since the five topological properties of the nodes also serve as positive indicators, they are ranked in ascending order. Employing the same computation method, the information entropy and weight for the five topological properties of nodes are calculated. These results are provided in Supplementary Table 5-1. The final graded comprehensive topological properties for nodes in subnetwork 2 are presented in Supplementary Table 5-1.

At site T, the Walktrap algorithm divided the correlation network into 37 subnetworks. The top eight subnetworks with the highest number of nodes (microbes) are subnetworks “8”, “4”, “2”, “3”, “1”, “9”, “11” and “7”. Then, the RSR-EWM evaluation algorithm is used to score the comprehensive topological properties of these subnetworks, with the highest ranking being “8”, which contains 25 nodes, 105 edges, a graph diameter of 2.499, a graph density of 0.350, etc. (Supplementary Table 5-1). Next, we comprehensively scored five topological property indicators for each of the 25 nodes in subnetwork "8" and found that *Rothia*, *Enterobacter*, *Streptomyces*, *Prevotella, Phyllobacterium and Gemella* were the top four highest scoring bacteria (Supplementary Table 5-1 and Supplementary figure 1), which were the central bacteria in subnetwork “8”. Using the same pipeline, the central bacteria of other subnetworks at the site of T and other oral sites could also be determined.

**Evaluating and scoring of microbial subnetworks in the Saliva (S) Region**

1. The original evaluation matrix is constructed based on the scores of the ten indicators for the five subnetworks as shown in Supplementary Table 4-4. The matrix is presented below:

$$X=\left[ \begin{matrix} 32 & 68 & 4.194 & 0.137 & 4.250 & 1 & 1 & 0.331 & 0.466 & 2.185 \\ 24 & 67 & 4.137 & 0.243 & 5.583 & 1 & 1 & 0.472 & 0.237 & 1.747 \\ 12 & 19 & 4.077 & 0.288 & 3.167 & 1 & 1 & 0.476 & 0.263 & 1.772 \\ 9 & 11 & 3.323 & 0.306 & 2.444 & 1 & 1 & 0.250 & 0.252 & 1.690 \\ 5 & 10 & 0.969 & 1.000 & 4.000 & 4 & 4 & 1.000 & -0.080 & 0.924 \end{matrix} \right]$$

2. Standardizing subnetwork topological property metrics. Scores are standardized using Equation (1), the detailed results are presented below:

$$Z=\left[ \begin{matrix} 1.000 & 1.000 & 1.000 & 0.000 & 0.686 & 0 & 0 & 0.331 & 1.000 & 0.000 \\ 0.724 & 0.985 & 0.983 & 0.122 & 1.000 & 0 & 0 & 0.472 & 0.667 & 0.319 \\ 0.310 & 0.258 & 0.965 & 0.175 & 0.431 & 0 & 0 & 0.476 & 0.705 & 0.301 \\ 0.207 & 0.136 & 0.742 & 0.195 & 0.261 & 0 & 0 & 0.250 & 0.689 & 0.361 \\ 0.069 & 0.121 & 0.043 & 1.000 & 0.627 & 1 & 1 & 1.000 & 0.207 & 0.920 \end{matrix} \right]$$

3. Rank $R_{ij}$​ of each metric of subnetwork topological properties is calculated. Among the ten network topological property indicators, nine positive metrics are arranged in ascending order. The final indicator, “average path length,” is negative metrics and arranged in descending order. Identical values for the same metric are given average ranks, producing the rank matrix $R$. The results are provided below:

$$R=\left[ \begin{matrix} 7 & 7 & 13 & 1 & 7 & 1 & 1 & 3 & 8 & 1 \\ 6 & 6 & 12 & 2 & 8 & 1 & 1 & 4 & 5 & 3 \\ 5 & 5 & 11 & 3 & 5 & 1 & 1 & 5 & 7 & 2 \\ 4 & 4 & 10 & 4 & 4 & 1 & 1 & 2 & 6 & 4 \\ 3 & 3 & 3 & 7 & 6 & 3 & 3 & 6 & 3 & 11 \end{matrix} \right]$$

4. Weights are determined using EWM. Based on Equation (4), the information entropy $e_{j}$ and entropy weight $W_{j}$ for ten evaluation metrics are ascertained, with outcomes present in Supplementary Table 5-2.
5. Determine the value of WRSR. Using Equation (5), the $WRSR$ for the five subnetworks is computed, with results provide below:

$$WRSR=\left[ 0.462, 0.433, 0.392, 0.324, 0.379 \right]$$

6. Distribution statistics of $WRSR$. In this step, the frequency, cumulative frequency, average rank, downward cumulative rating, and probability unit are tabulated in Supplementary Table 5-2. A higher rank indicates a better evaluation of the subject.
7. With the $Probit$ values corresponding to cumulative frequencies as the independent variable and $WRSR$ values as the dependent variable, the regression equation is computed using the least squares method: $WR\hat{S}R=-0.1071+0.0850\times Probit$，$R^{2}=0.939, F=92.06, P=7.33e-5, df=6$.
8. The topological properties of the subnetworks are ranked based on $Probit$ and $WR\hat{S}R$, with results presented in Supplementary Table 5-2.

**Evaluating and scoring of central node microbes at Saliva(S) Region**

Similar to subnetworks' composite topological properties, we employ an integrated rank-sum ratio–entropy weight evaluation model to evaluate the comprehensive topological attributes of all nodes within subnetworks 1. This decision is based on the scores of five indicators for nodes within the excellent subnetworks 1, as outlined in Supplementary material 2. Since the five topological properties of the nodes also serve as positive indicators, they are ranked in ascending order. Employing the same computation method, the information entropy and weight for the five topological properties of nodes are depicted in Supplementary Table 5-2. The final graded comprehensive topological properties for nodes in subnetworks 1 are presented in Supplementary Table 5-2.

At the site of S, Walktrap algorithm divides the correlation network into 24 subnetworks. The top five subnetworks with the highest number of nodes (microbes) are subnetwork “1”, “5”, “4”, “2” and “16”. Then, the RSR-EWM evaluation algorithm is used to score the comprehensive topological properties of these subnetworks, with the highest ranking being “1”, which contains 32 nodes, 68 edges, graph diameter 4.194, graph density 0.137, etc. (Supplementary Table 5-2). Next, we comprehensively score five topological property indicators for each of the 32 nodes in subnetwork "1" and find that *Veillonella*, *Granulicatella*, *Haemophilus* is the top three highest scoring bacteria (Supplementary Table 5-2), which is the central bacteria in subnetwork “1”.

**Evaluating and scoring of microbial subnetworks and central node microbes at the Buccal Mucosa (B) Region**

At the site of B, Walktrap algorithm divides the correlation network into 35 subnetworks. The top seven subnetworks with the highest number of nodes (microbes) are subnetwork “6”, “4”, “21”, “2”, “3”, “17” and “5”. Then, the RSR-EWM evaluation algorithm is used to score the comprehensive topological properties of these subnetworks, with the highest ranking being “4”, which contains 52 nodes, 222 edges, graph diameter 3.342, graph density 0.167, etc. (Supplementary Table 5-3). Next, we comprehensively score five topological property indicators for each of the 52 nodes in subnetwork "4" and find that *Actinomyces*, *Veillonella*, *Campylobacter* is the top three highest scoring bacteria (Supplementary Table 5-3), which is the central bacteria in subnetwork “4”.

**Evaluating and scoring of microbial subnetworks and central node microbes at the** **Gingival Crevicular Fluid (GCF) Region**

At the site of GCF, Walktrap algorithm divides the correlation network into 25 subnetworks. The top five subnetworks with the highest number of nodes (microbes) are subnetwork “2”, “3”, “5”, “10” and “6”. Then, the RSR-EWM evaluation algorithm is used to score the comprehensive topological properties of these subnetworks, with the highest ranking being “2”, which contains 21 nodes, 40 edges, graph diameter 5.783, graph density 0.190, etc. (Supplementary Table 5-4). Next, we comprehensively score five topological property indicators for each of the 21 nodes in subnetwork "2" and find that *Streptococcus* and *Neisseria* are the top two highest scoring bacteria (Supplementary Table 5-4), which is the central bacteria in subnetwork “2”.

**Evaluating and scoring of microbial subnetworks and central node microbes at the** **Dental Plaque (P) Region**

At the site of P, Walktrap algorithm divides the correlation network into 29 subnetworks. The top six subnetworks with the highest number of nodes (microbes) are subnetwork “4”, “2”, “3”, “1”, “5” and “9”. Then, the RSR-EWM evaluation algorithm is used to score the comprehensive topological properties of these subnetworks, with the highest ranking being “2”, which contains 27 nodes, 60 edges, graph diameter 4.144, graph density 0.171, etc. (Supplementary Table 5-5). Next, we comprehensively score five topological property indicators for each of the 27 nodes in subnetwork "2" and find that *Abiotrophia*, *Lachnospira* and *Porphyromonas* are the top three highest scoring bacteria (Supplementary Table 5-5), which is the central bacteria in subnetwork “2”.

**Reference**

1 Pons, P. & Latapy, M. in *Computer and Information Sciences-ISCIS 2005: 20th International Symposium, Istanbul, Turkey, October 26-28, 2005. Proceedings 20.* 284-293 (Springer).

2 R Core Team, R. R: A language and environment for statistical computing. (2013).

3 Brandes, U. *et al.* On modularity clustering. *IEEE transactions on knowledge and data engineering* **20**, 172-188 (2007).

4 Clauset, A., Newman, M. E. & Moore, C. Finding community structure in very large networks. *Physical review E* **70**, 066111 (2004).

5 Newman, M. E. & Girvan, M. Finding and evaluating community structure in networks. *Physical review E* **69**, 026113 (2004).

6 Junker, B. H. & Schreiber, F. *Analysis of biological networks*. (John Wiley & Sons, 2011).

7 White, D. R. & Harary, F. The cohesiveness of blocks in social networks: Node connectivity and conditional density. *Soc. Method.* **31**, 305-359 (2001).

8 Watts, D. J. & Strogatz, S. H. Collective dynamics of ‘small-world’networks. *Nature* **393**, 440-442 (1998).

9 Freeman, L. C. A set of measures of centrality based on betweenness. *Sociometry*, 35-41 (1977).

10 Poudel, R. *et al.* Microbiome networks: a systems framework for identifying candidate microbial assemblages for disease management. *Phytopathology* **106**, 1083-1096 (2016).

11 Sabidussi, G. The centrality index of a graph. *Psychometrika* **31**, 581-603 (1966).

12 Bonacich, P. Power and centrality: A family of measures. *American journal of sociology* **92**, 1170-1182 (1987).

13 Ruhnau, B. Eigenvector-centrality—a node-centrality? *Social networks* **22**, 357-365 (2000).

14 Bonacich, P. Factoring and weighting approaches to status scores and clique identification. *Journal of mathematical sociology* **2**, 113-120 (1972).

15 Wang, Q. & Cole, J. R. Updated RDP taxonomy and RDP Classifier for more accurate taxonomic classification. *Microbiology Resource Announcements* **13**, e01063-01023 (2024).

16 Myers, J. L., Well, A. & Lorch, R. F. *Research design and statistical analysis*. (Routledge, 2010).
