# Supplementary material for: MNetClass: a control-free microbial network clustering framework for identifying central subcommunities across ecological niches
Source: mSystems. 2025 Nov 13;10(12):e00989-25. doi: 10.1128/msystems.00989-25 (PMC12710344; doi:10.1128/msystems.00989-25)
Supplement: Legends — Supplemental figure legends. [file msystems.00989-25-s0006.docx]

**Supplementary Figure 1**

Key subnetworks and central microbes at the tongue (T) identified using the Walktrap algorithm and the integrated rank-sum ratio entropy weight evaluation model (RSR-EWM).

**Supplementary Figure 2**

Key subnetworks and central microbes at the buccal mucosa (B) identified using the Walktrap algorithm and the integrated rank-sum ratio entropy weight evaluation model (RSR-EWM).

**Supplementary Figure 3**

Key subnetworks and central microbes at the gingival crevicular fluid (GCF) identified using the Walktrap algorithm and the integrated rank-sum ratio entropy weight evaluation model (RSR-EWM).

**Supplementary Figure 4**

Key subnetworks and central microbes at the dental plaque (P) identified using the Walktrap algorithm and the integrated rank-sum ratio entropy weight evaluation model (RSR-EWM).
